# *Tnni3k* alleles influence ventricular mononuclear diploid cardiomyocyte frequency

**DOI:** 10.1371/journal.pgen.1008354

**Published:** 2019-10-07

**Authors:** Peiheng Gan, Michaela Patterson, Alexa Velasquez, Kristy Wang, Di Tian, Jolene J. Windle, Ge Tao, Daniel P. Judge, Takako Makita, Thomas J. Park, Henry M. Sucov

**Affiliations:** 1 Department of Regenerative Medicine and Cell Biology, Medical University of South Carolina, Charleston, South Carolina, United States of America; 2 Department of Medicine, Division of Cardiology, Medical University of South Carolina, Charleston, South Carolina, United States of America; 3 Department of Stem Cell Biology and Regenerative Medicine, University of Southern California Keck School of Medicine, Los Angeles, California, United States of America; 4 Department of Cell Biology, Neurobiology and Anatomy, and Cardiovascular Center, Medical College of Wisconsin, Milwaukee, Wisconsin, United States of America; 5 Department of Pathology and Laboratory Medicine, Tulane University School of Medicine, New Orleans, Louisiana, United States of America; 6 Department of Human and Molecular Genetics, Virginia Commonwealth University, Richmond, Virgina, United States of America; 7 Darby Children’s Research Institute, Department of Pediatrics, Medical University of South Carolina, Charleston, South Carolina, United States of America; 8 Laboratory of Integrative Neuroscience, Department of Biological Sciences, University of Illinois at Chicago, Chicago, Illinois, United States of America; Indiana University Purdue University at Indianapolis, UNITED STATES

## Abstract

Recent evidence implicates mononuclear diploid cardiomyocytes as a proliferative and regenerative subpopulation of the postnatal heart. The number of these cardiomyocytes is a complex trait showing substantial natural variation among inbred mouse strains based on the combined influences of multiple polymorphic genes. One gene confirmed to influence this parameter is the cardiomyocyte-specific kinase *Tnni3k*. Here, we have studied *Tnni3k* alleles across a number of species. Using a newly-generated kinase-dead allele in mice, we show that Tnni3k function is dependent on its kinase activity. In an in vitro kinase assay, we show that several common human TNNI3K kinase domain variants substantially compromise kinase activity, suggesting that TNNI3K may influence human heart regenerative capacity and potentially also other aspects of human heart disease. We show that two kinase domain frameshift mutations in mice cause loss-of-function consequences by nonsense-mediated decay. We further show that the *Tnni3k* gene in two species of mole-rat has independently devolved into a pseudogene, presumably associated with the transition of these species to a low metabolism and hypoxic subterranean life. This may be explained by the observation that *Tnni3k* function in mice converges with oxidative stress to regulate mononuclear diploid cardiomyocyte frequency. Unlike other studied rodents, naked mole-rats have a surprisingly high (30%) mononuclear cardiomyocyte level but most of their mononuclear cardiomyocytes are polyploid; their mononuclear diploid cardiomyocyte level (7%) is within the known range (2–10%) of inbred mouse strains. Naked mole-rats provide further insight on a recent proposal that cardiomyocyte polyploidy is associated with evolutionary acquisition of endothermy.

## Author summary

Embryonic cardiomyocytes have one diploid nucleus (like most cells of the body), but most adult cardiomyocytes are polyploid. Most adult cardiomyocytes are also post-mitotic and nonregenerative, and as a result, heart injury (such as from a heart attack) is followed by scarring and impaired function rather than by regeneration. A subset of cardiomyocytes in the adult heart remains mononuclear diploid, and recent evidence indicates that this subpopulation has proliferative and regenerative capacity. Our previous work in mice showed that the percentage of this cell population in the adult heart is a complex trait subject to the combined influence of a number of polymorphic genes. One gene that influences variation in this trait is a kinase gene known as *Tnni3k*. This study addresses the consequences of a number of *Tnni3k* alleles, both newly engineered in mice and naturally occurring in a number of species, including human and mole-rat, and studied at the phenotypic and biochemical level. These results provide insight into inter- and intra-species variation in the cardiomyocyte composition of the adult heart, and may be relevant to understanding heart regenerative ability in humans and across other species.

## Introduction

The ability of the heart to regenerate after injury is an indication of the proliferative capacity of its cardiomyocytes (CMs). In all species with hearts, the embryonic heart grows during development by CM proliferation, with a rate that peaks and then declines to minimal levels as the proper number of CMs is reached. Long after the phase of rapid embryonic CM proliferation is past, zebrafish and newts throughout life are able to regenerate their hearts after injury by reactivating CM proliferation [1–3]. In the early mammalian neonate, heart injury also induces robust reactivation of CM proliferation that leads to efficient regeneration, as observed in rat [4], mouse [5], pig [6, 7], and human [8, 9]. However, this response is mostly lost in mammals soon after birth (in mice, during the first postnatal week [5]). This has major clinical significance. A common cause of human heart injury is atherosclerotic coronary artery occlusion leading to myocardial ischemia or infarction followed by CM death in the impacted region. For lack of adequate CM proliferation and regeneration, the aftermath of adult heart injury is scar formation and permanent loss of myocardial function. Diminished heart function can progress to heart failure, which is among the largest impacts on U.S. healthcare costs [10]. There is still grossly insufficient insight as to why and how the mammalian heart becomes nonregenerative in the early postnatal period, and lack of understanding of this process is a critical barrier to progress in improving human health and adult post-injury outcomes.

CMs become polyploid coincident with the loss of their proliferative ability. In all species, all CMs are mononuclear and diploid (like most cells of the body) during embryonic development. In mammals shortly after birth (during the first postnatal week in mice), there is a final CM entry into the cell cycle (i.e., S phase DNA replication) but with arrest either before or after karyokinesis but prior to completion of cytokinesis, such that most CMs at this time become polyploid. In mice and rats and believed for all rodents, most CMs become binucleated [11–14], whereas in humans there is a greater frequency of mononuclear CMs with tetraploid nuclei [15–17]. CMs that are mononuclear tetraploid or CMs that are binucleated with two diploid nuclei are both referred to as being polyploid; higher levels of CM polyploidy, both in number of nuclei per CM and in the ploidy of each CM nucleus, also occur. All of these are distinguished from mononuclear diploid CMs. CM polyploidization is thought to occur through an interruption in the cell cycle machinery that controls karyokinesis and/or cytokinesis, and while there are initial insights [18, 19], relatively little is known of the mechanics of this process. Only a limited fraction of neonatal CMs complete cytokinesis at this time to form new mononuclear diploid CMs. Thereafter, cell cycle activity is mostly nonexistent, and the CM composition (the ratio of mononuclear diploid to polyploid CMs) of the mammalian heart remains constant through adulthood. In zebrafish and newts, polyploidization does not occur, and these species retain the capacity for highly efficient CM proliferation and regeneration throughout life. Thus, from a comparison of multiple species and life stages, there is a precise correlation between the abundance of mononuclear diploid CMs and competence to support CM proliferation for embryonic heart growth or post-injury heart regeneration.

Two recent studies move the relationship of CM ploidy and regeneration from correlation to causation. In mouse, the level of ventricular mononuclear diploid CMs in the normal adult heart is typically measured as approx. 2%. In our work [20], rather than assuming this to be a fixed feature of the adult heart, we showed that the percentage of these CMs in the adult heart is surprisingly variable between inbred mouse strains, reaching as high as 10% in some. We showed that strains with more of these had better regeneration at the functional and cellular level after adult heart injury. By genome-wide association, we identified one gene, a CM-specific kinase named *Tnni3k*, as having a naturally-occurring loss-of-function variant that influences mononuclear CM percentage, and there are clearly other polymorphic genes that also contribute to variation in this trait. The *Tnni3k* gene is functional in C57BL/6J mice and in many other strains with lower mononuclear CM level, whereas many inbred strains with higher mononuclear CM content are homozygous for the natural loss-of-function allele. These strains all appear normal and healthy and have been maintained for decades, showing that the *Tnni3k* gene is not required for life. Engineered knockout of this one gene in the C57BL/6J strain background resulted in the predicted increase in mononuclear diploid CM percentage, and a consequent improvement in CM cellular regeneration after adult heart injury. Reciprocally, transgenic overexpression of *Tnni3k* in zebrafish increased polyploidy and impaired adult zebrafish heart regeneration.

A zebrafish study from a different lab [21] reached a similar conclusion. In this analysis, transient transgenic expression of a dominant negative version of the cytokinesis component Ect2 caused a high degree of CM polyploidy, and much later (after the dominant negative protein was no longer present, but CMs were still polyploid), adult fish were consequently not able to regenerate effectively. The significance of the two studies is in their use of direct experimental approaches, rather than observational correlations, to conclude that mononuclear diploid CMs can be proliferative and regenerative, whereas the polyploid CM state is at least mostly nonproliferative and nonregenerative. In most mammalian species and in most individuals within a species, there might be relatively few mononuclear diploid CMs, but in some cases there could be more, and perhaps many more, of these potentially regenerative CMs.

TNNI3K in humans is 835 amino acids long, with an extended amino-terminal ankyrin repeat domain comprising the first half of the protein, followed by a kinase domain, and a serine-rich carboxy-terminal domain. In several human pedigrees, mutations in the *TNNI3K* gene have been associated with conduction system disease (of various manifestations) and dilated cardiomyopathy. One of these is a mutation at the splice donor sequence following exon 4 (of a total of 25 exons) that presumably terminates the open reading frame at a downstream intronic stop codon [22]. The others are point mutations in the kinase domain (Thr539Ala and Gly526Asp) [23, 24] or in the C-terminal domain (Glu768Lys) [25]. These pathogenic mutations are rare (i.e., are not found in the ExAC compilation [26] of human whole exome sequence), and are disease-associated as heterozygous alleles.

Other than *Tnni3k*, no gene in any species has yet been identified to have natural variants that influence CM polyploidy. *Tnni3k* therefore provides a unique opportunity to understand the genetic basis of CM polyploidization in a way that has natural correlates and potentially also influences human heart disease.

## Results

### New *Tnni3k* alleles in C57BL/6J mice subject to nonsense-mediated decay

The natural mouse *Tnni3k* variant [27] that revealed this gene to be relevant to mononuclear CM frequency in our prior genome-wide association is a splice site mutation at position +9 of intron 19 (position 154875123 of NC_000069.6). This variant shifts the exon 19 splice donor by 4nt, thereby creating a frameshift after Gly625 that terminates one amino acid later. By nonsense-mediated decay, this frameshifted transcript is degraded. This variant allele is therefore a loss-of-function mutation. The wild-type functional allele is present in C57BL/6J mice.

**Fig 1:**
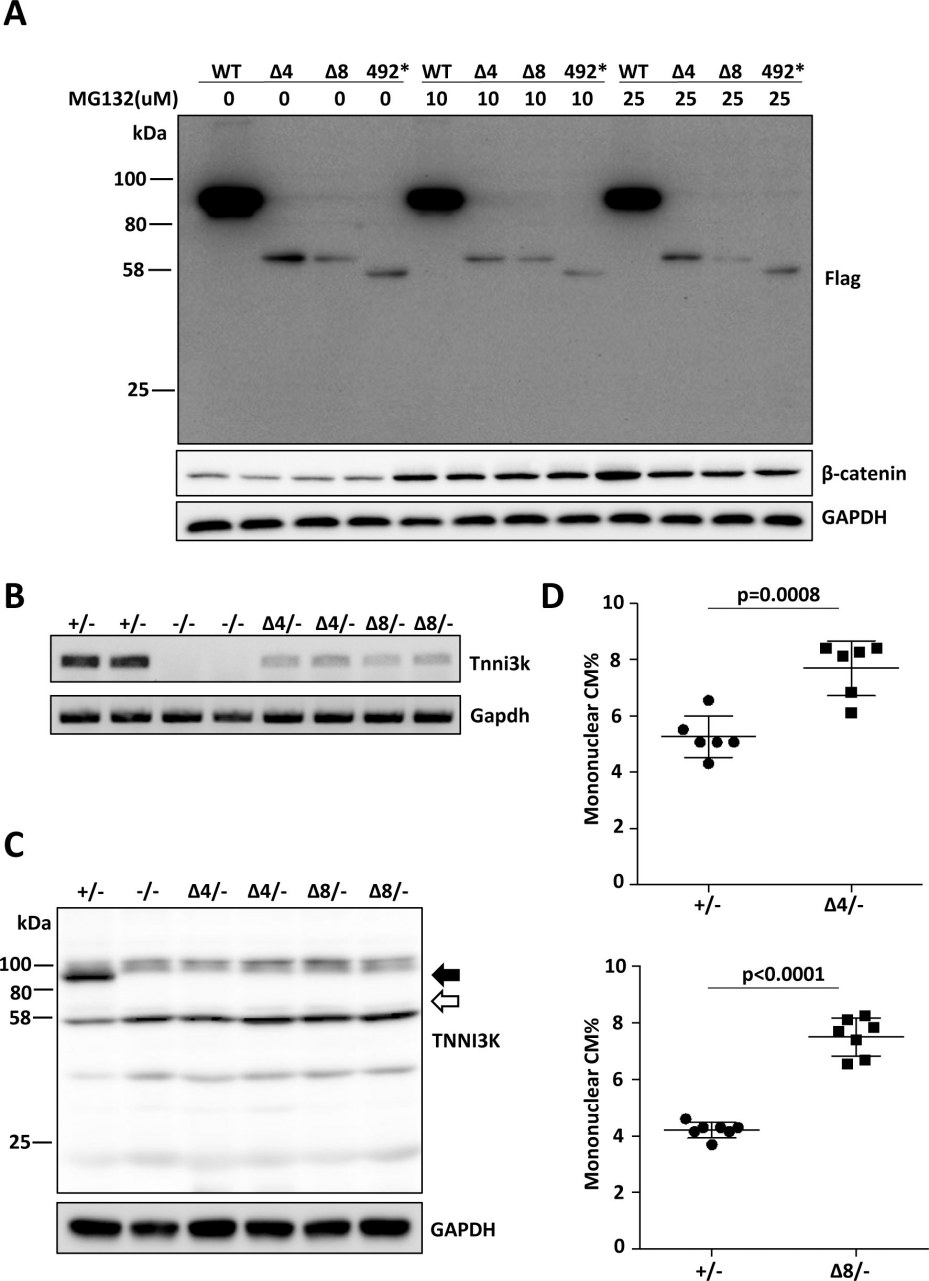
*Tnni3k* deletion mutants cause nonsense-mediated mRNA decay. **A.** Western blot of protein extracted 293 cells transfected with constructs to express wild-type full-length mouse Tnni3k (WT), the 4bp or 8bp deletion mutant variants (Δ4 and Δ8), and a truncation mutation at position 492 corresponding to the premature stop codon observed in *H*. *glaber*. All constructs were based on the mouse protein, and all carried a Flag epitope at the N-terminus. Transfected protein was detected by anti-Flag antibody. MG132 was added at the concentrations indicated to test for proteasomal degradation. Gapdh was used as a loading control. **B**. Semi-quantitative RT-PCR analysis of *Tnni3k* transcript abundance in ventricular tissue from adult mice (2 of each genotype) of the indicated genotypes. **C.** Western blot to detect Tnni3k protein in ventricular lysate from adult mice of the indicated genotypes. The filled arrow indicates full-length Tnni3k (93kDa), the open arrow indicates the expected size of the Δ4 and Δ8 truncated proteins (62kDa) if they were stably expressed. **D.** Ventricular mononuclear CM% in adult hearts of the indicated genotypes; in both evaluations, littermate +/- control mice were used as a reference for Δ4/- or for Δ8/- experimental mice.

In the course of making other CRISPR-mediated alleles in C57BL/6J mice, we recovered two lines in which small deletions, one of 4bp and one of 8bp, were introduced into exon 16 (S1 Fig). For simplicity, these alleles are named here as Δ4 and Δ8. Both result in frameshifts after Ser532 and in termination downstream after 29 and 16 ectopic codons, respectively, both well before the end of the wild-type open reading frame in exon 25. We therefore expected that transcripts derived from these alleles, like the natural variant allele described above, would be degraded by nonsense-mediated decay. When overexpressed in transfected 293 cells, these transcripts resulted in only a very low level of truncated proteins (Fig 1A). To negate the possibility that low protein level was a manifestation of proteasomal degradation, we treated transfected 293 cells with the proteasomal inhibitor MG132. Beta-catenin, which is known to be regulated by proteasomal degradation [28], served as the positive control, and was increased after MG132 treatment. The level of the wild-type full length Tnni3k protein was not influenced by MG132, indicating that the abundance of this protein is not regulated by proteasomal degradation. Similarly, the low levels of the truncated Δ4 and Δ8 proteins were not impacted by MG132. This implicates nonsense-mediated transcript decay or nonproteasomal degradation as the basis for the low protein levels observed in transfected 293 cells.

Founder mice heterozygous for the deletion alleles were crossed to wild-type C57BL/6J partners to confirm germline establishment of the allele, and heterozygous F1 mice then crossed to C57BL/6J-inbred *Tnni3k* homozygous null mice (derived in our previous study [20]) to generate littermate mice that were used for analysis. By this approach, any off-target mutation co-introduced by CRISPR manipulation would at most be heterozygous but not homozygous, and a uniform C57BL/6J background was maintained. *Tnni3k* Δ4/- and Δ8/- mice, like null (-/-) mice, were externally normal. Ventricular lysates prepared from adult hearts demonstrated a substantially reduced level of mRNA (Fig 1B) and no detectable Tnni3k protein at the expected (62kDa) size range (Fig 1C; note that the antibody used here was targeted to amino acids 187–403, so would be able to detect either truncated protein if present). In comparison to transfected 293 cells, where a low level of truncated protein was observed (Fig 1A), the absence of detectable protein in heart tissue may be explained by one or more of several potential causes: nonsense-mediated decay may be more efficient in cardiomyocytes, or the lower level of gene expression in vivo might not saturate the decay machinery as may occur with high expression in transfected 293 cells, or the anti-Flag antibody may be more sensitive to low protein levels in Western blots than the anti-Tnni3k antibody.

Following collagenase digestion and preparation of single cell suspensions, adult hearts were evaluated for ventricular mononuclear CM content. In our previous analysis of a loss-of-function *Tnni3k* null allele in C57BL/6J-inbred mice [20], we observed that the mononuclear CM frequency of wild-type (+/+) and heterozygous (+/-) hearts was the same, and was increased in homozygous null (-/-) mice. The same increase was observed in ventricles from Δ4/- and Δ8/- mice as in -/- mice (Fig 1D). Although the nuclear ploidy of the mononuclear CM subpopulation of these mice was not directly measured, as shown below, control and *Tnni3k* null mice on an inbred C57BL/6J background have the same percentage of diploid nuclei in their mononuclear CM subpopulation. Thus, mononuclear CM number but not nuclear ploidy is affected by *Tnni3k* gene status, such that mononuclear CM frequency is a suitable surrogate for mononuclear diploid CM frequency in the C57BL/6J background. The Δ4 and Δ8 alleles therefore behave as loss-of-function alleles that increase mononuclear CM content, just as inferred for the natural intronic splice site mutant allele. The principle advance afforded by this analysis lies in the derivation of the two frameshift alleles on an inbred C57BL/6J background. Thus, rather than comparing mononuclear CM content between mice of different strain backgrounds carrying different *Tnni3k* alleles, here we confirm on a single strain background that a transcript encoding a prematurely truncated *Tnni3k* open reading frame has the same phenotypic effect as a full null allele.

### Analysis of *Tnni3k* and CM composition in naked mole-rats, a poikilothermic subterranean rodent

*Tnni3k* expression in mammals is mostly restricted to the heart, likely in part through a Mef2 binding site in the promoter region [29]. A *Tnni3k* gene is present in all mammals, and recognizable *Tnni3k* homologs exist in numerous invertebrate species among both the deuterostome and protostome subdivisions of the animal kingdom, including those not thought to have a heart (e.g., sea urchins; S1 Table). The ancestral *Tnni3k* gene was therefore present early in animal evolution; it is not known when cardiac-restricted expression evolved.

**Fig 2:**
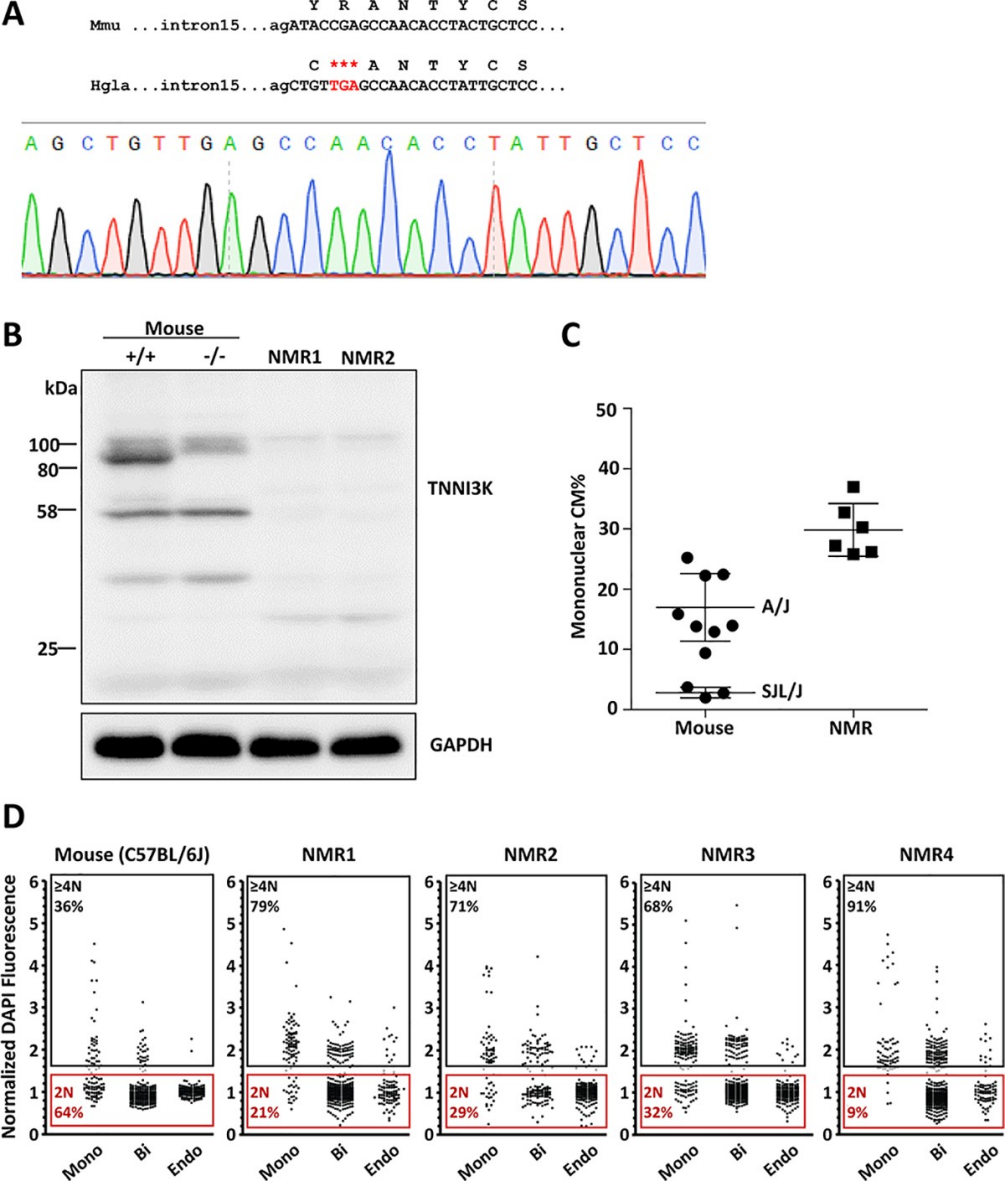
The *Tnni3k* gene in naked mole-rats (*H*. *glaber*). **A.** Genomic DNA sequence at the end of intron 15 and beginning of exon 16 and deduced protein coding sequence (in 1 letter code above) in mouse (Mmu) and in *H*. *glaber* (Hgla). The in-frame premature stop codon is indicated in red type, and lies at position 22288559–561 (reverse complement) of NW_004624742.1 and at 1144837–839 (reverse complement) of NW_004629930.1. The sequence trace confirms the accuracy of the *H*. *glaber* genomic sequence. **B.** Western blot to detect Tnni3k; the blot contains ventricular protein from one wild-type (WT) C57BL/6 mouse, one homozygous null (-/-) mouse, and two individual *H*. *glaber* animals. **C.** Ventricular mononuclear CM% in adult hearts of two inbred mouse strains (SJL/J and A/J), which represent the lowest and highest known values of mononuclear CM% among common inbred strains, and in adult *H*. *glaber*. Data for SJL/J and A/J mice are from our previous evaluation [20] and are shown here for reference. **D.** Evaluation of nuclear ploidy in single cell preparations from one C57BL/6J mouse (at left) and four naked mole-rats, as measured by DAPI fluorescence. Values shown represent fluorescence intensity per nucleus from mono- and bi-nucleated cardiomyocytes and endothelial cells, normalized to the median intensity of endothelial cell nuclei; a normalized value of 1.0 is assumed to be the median signal from a diploid nucleus. Each dot is one measured nucleus. Numerical quantitations of 2N and ≥4N status as shown are only for the mononuclear CM group; compiled numerical data for both CM subpopulations are shown in S5 Table.

From a survey of public database *Tnni3k* gene sequences across mammalian species, we found that the gene for the naked mole-rat (*Heterocephalus glaber*) contained a premature stop codon at the position corresponding to mouse Arg492 in exon 16 early in the encoding portion of the kinase domain. This sequence was observed in two independent *H*. *glaber* genomic sequence assemblies (NW_004624742.1 and NW_004629930.1) and was confirmed by direct genomic sequencing (Fig 2A) in both of two *H*. *glaber* animals (albeit from the same colony and so possibly related). Importantly, the stop codon was homozygous in both. Thus, this is unlikely to be a minor variant and more likely represents the reference sequence of the *H*. *glaber* species. We modeled a premature stop codon in the mouse *Tnni3k* gene at position 492, and in transfected 293 cells observed that this resulted in a low level of truncated protein, exactly equivalent in behavior to what was observed with the Δ4 and Δ8 frameshift transcripts (Fig 1A). Therefore, the *H*. *glaber* sequence at this position is predicted by itself to cause nonsense-mediated decay and result in absence of protein expression in vivo, just as with the mouse Δ4 and Δ8 alleles.

The available genomic sequence of *H*. *glaber Tnni3k* implied two additional premature stop codons within the kinase domain coding region, one associated with a frameshift mutation in the equivalent of mouse exon 20 and the other a nonsense codon in the equivalent of mouse exon 21, and both were confirmed by direct genomic sequencing (panels A-B of S2 Fig). A full comparison of database genomic sequence for the *M*. *musculus* and *H*. *glaber Tnni3k* genes demonstrated a very high degree of conservation, and notably in the first 15 exons encoding the amino-terminal ankyrin repeat domains and the beginning of the kinase domain. With allowance for one abnormal splice acceptor sequence (GG rather than AG preceding exon 13), a continuous open reading frame with high homology to the mouse protein could be assembled computationally from these 5’ exons (S2 Table, panels C-D of S2 Fig). Although nonsense-mediated decay should degrade any transcript with a stop codon in any exon before the terminal exon, exceptions are known, and it therefore remained possible that the *H*. *glaber* gene might encode a protein with only ankyrin repeats that terminated at the stop codon corresponding to mouse Arg492 in the beginning of the kinase domain. Western blotting of *H*. *glaber* ventricular lysates with Tnni3k antibody (recognizing the N-terminal domain and the same as used above) did not reveal a band around the size predicted for a truncated protein (56kDa) (Fig 2B), although the antibody might not have been able to recognize the naked mole-rat protein sequence. Thus it remained uncertain if the *H*. *glaber* gene encodes a protein.

To resolve these questions, we amplified the *H*. *glaber* cDNA corresponding to mouse *Tnni3k* exons 1–15. No band corresponding to the expected full-length sequence (i.e., 1479 bp) was detected, but a smaller band of 1257 bp was amplified on two separate occasions using heart cDNA samples from two separate animals. These products were both sequenced and both found to be identical. The deduced cDNA (panel E of S2 Fig) includes a number of changes relative to the predicted transcript extracted from the genomic sequence assembly based on homology to the mouse gene (S2 Table). Most significantly, exon 2 was spliced to exon 6, bypassing the intervening potential exons 3–5. These three exons represent 310 bp of sequence, and their exclusion results in a frameshift that terminates the open reading frame after the 83rd encoded amino acid (the first 50 corresponding to the N-terminal mouse protein, the subsequent 33 being ectopic and resulting from the frameshift). Other observed changes included a 51nt readthrough of the exon 12 predicted splice donor (based on the mouse sequence) to employ a downstream splice donor sequence (intronic in mice) that was spliced to exon 14 and bypassed exon 13, and complete readthrough of the small intron 14 (79nt in *H*. *glaber*; the corresponding intron is 93nt in mouse) directly into exon 15. As noted above, the kinase domain premature stop codon that originally drew our attention to the *Tnni3k* gene in *H*. *glaber* is in exon 16. We also observed one mismatch between the cDNA sequence and the corresponding genomic sequence in the database, a G for A substitution in exon 6 (position 22318710 of NW_004624742.1) that may be a natural variant or may represent a sequencing error in the genomic assembly. The altered splicing pattern of the *H*. *glaber Tnni3k* gene as deduced from the cDNA sequence was predictable in one case: the database genomic sequence at the splice acceptor site preceding exon 13 was GG rather than AG (as noted above), explaining why this exon was skipped. However, all other intron junctions appeared normal in the genomic sequence through the first 15 exons (these genomic sequences were not directly confirmed by us). Despite the fairly high conservation between the mouse and naked mole-rat genomic sequences corresponding to each mouse exon, we conclude that the naked mole-rat *Tnni3k* gene does not encode any protein and may have devolved into a pseudogene. Nonetheless, the gene is still transcriptionally active in the heart, and generates a transcript that is predicted to mostly be degraded by nonsense-mediated decay.

In the genome sequence database (S3 Table), we found that the *Tnni3k* gene of another mole-rat species, the Damaraland mole-rat (*Fukomys damarensis*), also appeared to contain multiple frame shifts and premature stop codons. We directly sequenced exons 5 and 15 from one animal, and observed a frameshift and a premature stop codon in both exons (panel F of S2 Fig). Importantly, the *Tnni3k* gene frame-shifts, premature stop codons, and splice donor or acceptor mutations in *H*. *glaber* were not found in *F*. *damarensis*, and vice versa. The two mole-rat species diverged from each other approx. 25–30 million years ago, which is on the order of the divergence time between mouse and rat [30–33]. We conclude that the ancestral *Tnni3k* gene was present and functional in the common rodent ancestor, and then devolved independently in both *H*. *glaber* and *F*. *damarensis*. Possibly, this is an example of parallel evolution associated with the independent transition of both species to subterranean life. Alternatively, the last common ancestor of both species of mole-rat may have already acquired an initial loss-of-function mutation in the *Tnni3k* gene, perhaps associated with it becoming subterranean, with the gene then acquiring further mutations independently in each species after they became reproductively isolated.

Prior studies have reported *H*. *glaber* heart morphology and physiology [34, 35], although CM polyploidy had not been addressed. We conducted an analysis of ventricular CM composition in juvenile-adult stage naked mole-rats, using the same procedures as used for mouse heart analysis. Unexpectedly, we found that that 30% of the CM population was mononuclear (Fig 2C), which is substantially higher than any of our previously measured inbred mouse strains. Although the great majority of CMs in mice and rats are binucleated (e.g., 95% in C57BL/6J mice), human CMs are more commonly mononuclear tetraploid, such that a direct measurement of nuclear ploidy in naked mole-rat mononuclear CMs was worthwhile. Because fluorescence in situ hybridization probes for direct visualization of chromosome number (as we used previously for mouse [20]) are not available for *H*. *glaber*, we instead estimated nuclear ploidy in stained ventricular single cell suspensions by DAPI fluorescence intensity, normalized to the intensity of nuclei in Pecam1+ endothelial cells (which are presumed to be diploid unless in the process of cell division). This analysis (Fig 2D, S5 Table) indicated that the majority (77%) of naked mole-rat mononuclear CMs are polyploid (tetraploid or higher nuclear ploidy level), and that the prevalence of mononuclear diploid CMs in this species is therefore on the order of 7%. We of course do not know the extent of genetic variation or the range of mononuclear diploid CM frequency across the *H*. *glaber* species, so this assessment is a single data point based on only one group of animals. One conclusion from this analysis is that mononuclear tetraploid CMs can be a substantial fraction of ventricular CMs in rodents (e.g., for *H*. *glaber* CMs, 70% are binucleated, 23% are mononuclear tetraploid, 7% are mononuclear diploid), and that it cannot be assumed for all rodent species that most mononuclear CMs are diploid. While a 7% level of mononuclear diploid CMs is high compared to most mouse strains and to most other mammalian species that have been studied, this is less than in mouse strain A/J, which has the highest level of mononuclear diploid CMs (10%) of the inbred mouse strains that we have surveyed [20]. While an *H*. *glaber* substrain with a functional *Tnni3k* gene is not available for comparison, we suggest that absence of *Tnni3k* gene function in naked mole-rats may contribute to the moderately high level of mononuclear diploid CMs seen in this species, just as we have shown experimentally in mice.

### Tnni3k kinase activity regulates mononuclear cardiomyocyte frequency

By sequence, Tnni3k is a member of the MLK family of kinases, which have both tyrosine and serine/threonine phosphorylation activity, and is classified as a MAP3K. Although the protein has previously been shown to have autophosphorylation activity in vitro [25, 36, 37], no natural proteins that serve as kinase substrates in vivo have yet been verified. Furthermore, kinase activity in the context of establishment of the mononuclear diploid CM frequency cannot be assumed, as the protein could act solely via interactions with other proteins through its N- or C-terminal domains; this is not without precedent in other kinases [38].

**Fig 3:**
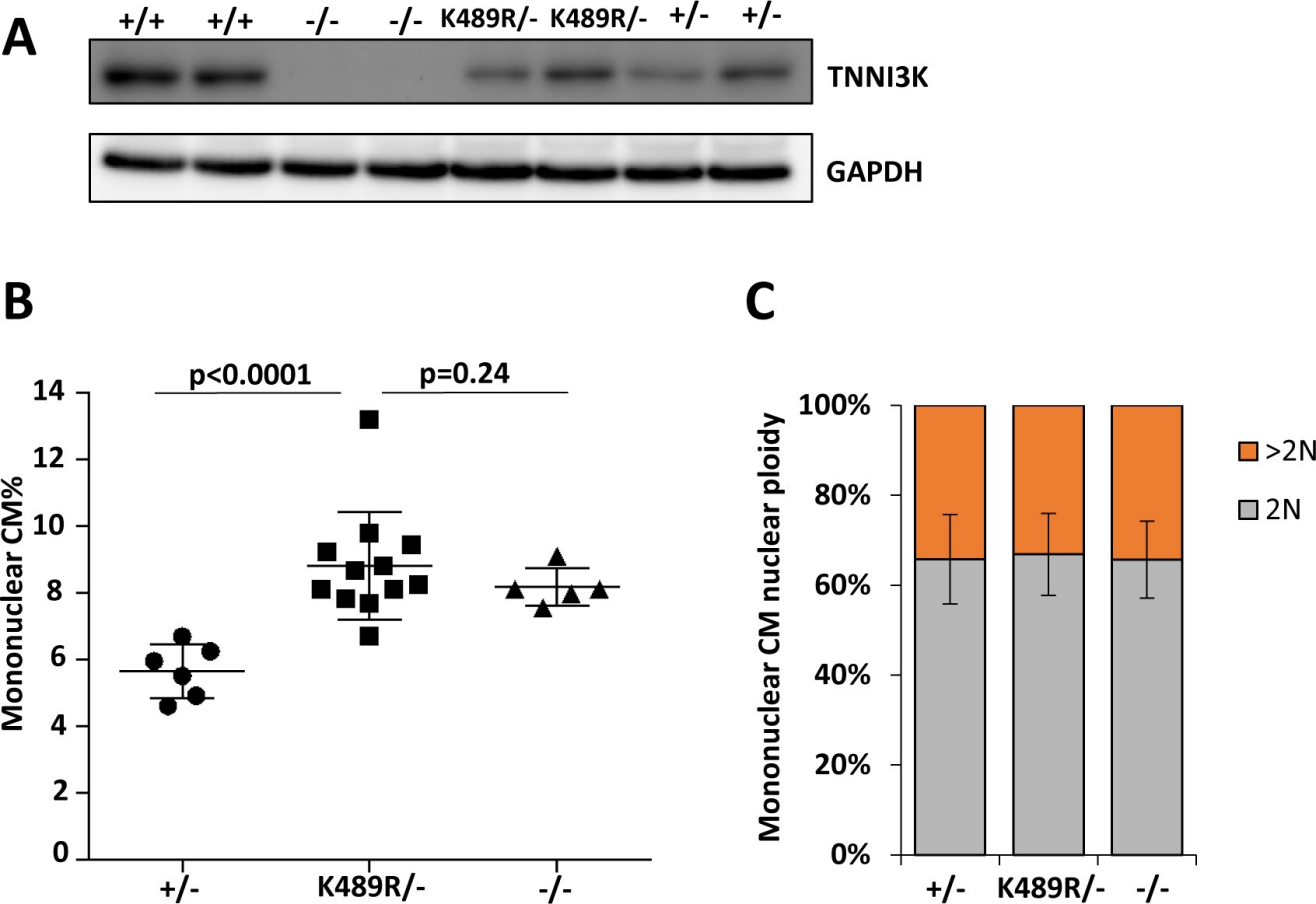
Consequences of a kinase-dead *Tnni3k* allele in mice. **A.** Western blot of Tnni3k protein from mice of the indicated genotypes, demonstrating that the K489R protein is stable in vivo. **B.** Ventricular mononuclear CM% in mice of the indicated *Tnni3k* genotypes; heterozygous (+/-) and null (-/-) mice were littermates of the K489R/- mice, but not of each other. **C.** Evaluation of nuclear ploidy specifically in the mononuclear CM subpopulation of mice of the indicated *Tnni3k* genotypes. See also S4 Fig.

To demonstrate the in vivo function of Tnni3k kinase activity, we created a kinase-dead mutant allele in C57BL/6J mice by CRISPR-mediated homologous replacement, changing the lysine codon at position 489 to arginine (AAA to AGA) in exon 15 (panel A of S3 Fig). This lysine sits in the ATP binding pocket, and among all kinases, a lysine in this position is almost ubiquitously conserved as it coordinates the terminal phosphate of ATP as it is transferred to a substrate. In all other studied kinases, mutation of this lysine to any other amino acid (including arginine) abolishes kinase activity. As previously shown [25, 37] and as reconfirmed here (see below), Tnni3k with the K489R mutation was stable when expressed in 293 cells, and in an in vitro kinase assay was devoid of autophosphorylation activity. A founder female mouse heterozygous for the K489R allele was crossed to wild-type C57BL/6J males to confirm germline establishment of the allele, and heterozygous F1 mice then crossed to C57BL/6J-inbred *Tnni3k* homozygous null mice to generate littermate mice that were used for analysis. Just as homozygosity of the *Tnni3k* null allele has no externally obvious consequence, mice in which the K489R allele and the null allele were combined were also not obviously impacted. Ventricular lysates from these mice contained the expected level of full-length protein (Fig 3A), confirming that the K489R point mutation does not alter protein stability in vivo.

Adult mouse hearts were evaluated for their percentage of ventricular mononuclear CMs. Mice with the kinase-dead K489R allele (K489R/-) had a significantly increased level of mononuclear CMs relative to heterozygous (+/-) mice and equivalent to the level in null (-/-) mice (Fig 3B), and equivalent also to Δ4/- and Δ8/- mice (Fig 1D; not compared in the same analysis). Direct measurement of nuclear ploidy in mononuclear CMs (Fig 3C, S4 Fig, S5 Table) showed no change across the +/-, K489R/-, and -/- genotypes. Thus, Tnni3k kinase activity regulates mononuclear diploid cardiomyocyte frequency. Because absence of Tnni3k protein has the same consequence as a normal level of the kinase-dead full-length protein, we also conclude that *Tnni3k* has no additional role through an independent function (e.g., as a scaffold for interaction with other proteins). The amino-terminal ankyrin repeat domain and the carboxy-terminal serine-rich domain may also be required to modulate Tnni3k’s kinase role in this context [36], but not in a manner that is independent of its kinase activity.

### Common human *TNNI3K* polymorphisms compromise TNNI3K kinase activity

The ExAC database of aggregated human whole exome sequence [26] indicates that *TNNI3K* loss-of-function alleles are moderately rare in the human population (0.3% of sequenced alleles). These include frameshifts, premature stops, and splice site mutations that presumably result in frameshifts. These variants are distributed over the entire length of the gene, with most therefore appearing in exons 5’ to the last large intron. Based on the rules of nonsense-mediated decay [39] and by extrapolation from the various *Tnni3k* alleles described above, we predict that these human variants would also be subjected to nonsense-mediated decay and would not encode any protein. Supporting this prediction, one of the disease-associated rare *TNNI3K* variants, a mutation in the splice donor sequence following exon 4, was measured to cause nonsense-mediated decay [22]. For the two most common loss-of-function variants in the ExAC database (a premature stop codon in exon 3 at position 65, and a 10bp insertion in exon 8 causing a frameshift at position 241; both positions based on NP_057062.1), one individual for each was identified in the ExAC database to be homozygous. No clinical or phenotypic information is available for these two individuals, but much as homozygous *Tnni3k* loss-of-function alleles in mice do not cause any externally obvious consequence, we predict these individuals to also be overtly normal. If our mouse studies of *Tnni3k* can be extended to human heart biology, we also predict these individuals to have a higher percentage of mononuclear diploid cardiomyocytes in their hearts.

**Fig 4:**
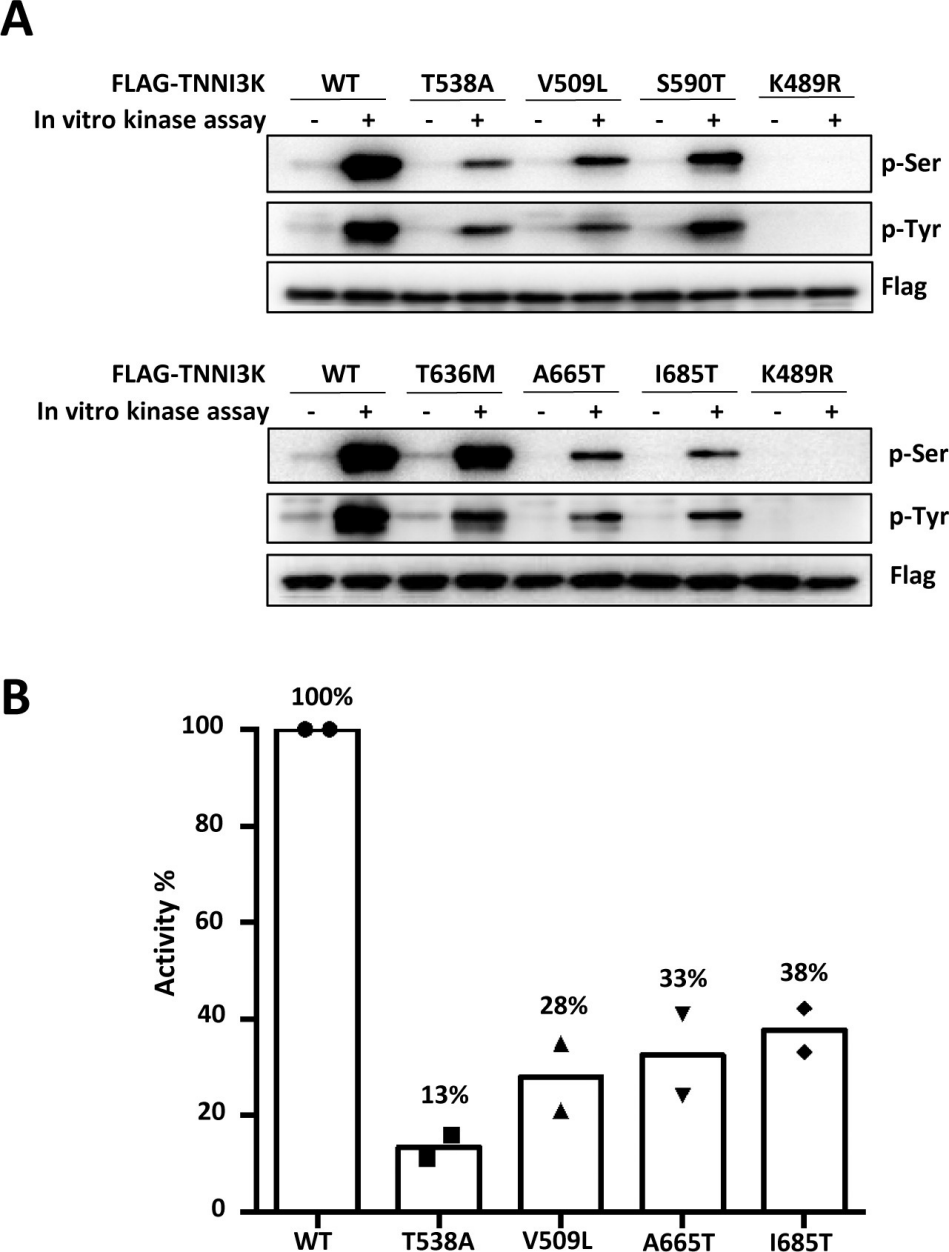
Consequences on in vitro kinase activity of common human *Tnni3k* kinase domain variants. **A.** Representative Western blot showing autophosphorylation on Ser and Tyr residues of wild-type and variant Tnni3k extracted from transfected 293 cells prior to (-) or following (+) incubation in an in vitro kinase reaction. Proteins were expressed with an N-terminal Flag epitope, which was detected separately as a loading control. **B.** Quantitation of kinase activity for the four indicated variants based on data shown in S5 Fig.

Compared to the loss-of-function alleles, a much larger number of nonsynonymous amino acid substitution variants of unknown significance occur in the human *TNNI3K* gene. Many of these lie in the kinase domain and therefore could influence kinase activity. We used the in vitro kinase assay mentioned above to assess the activity of the five most common such variants (S4 Table), engineered into the mouse protein. For comparison, a human disease-associated rare *TNNI3K* allele, Thr539Ala [23], was also engineered into the mouse full-length sequence, and the kinase-dead K489R mutant described above was also included in this analysis. All proteins were stable when expressed in 293 cells, as evident by detection of the Flag epitope at the N-terminus of each protein (Fig 4A). A summary compilation of the autophosphorylation kinase activity of each protein is shown in Fig 4B (numbering based on the mouse protein sequence, which is one fewer than the human sequence), and the primary data used for quantitation of kinase activity are shown in S5 Fig. Two of the human kinase domain variants (S591T and T637M, corresponding in Fig 4 to positions 590 and 636 in the mouse sequence, respectively) had little if any impact on kinase activity and were not further examined. However, three others (V510L, A666T, I686T) prominently compromised kinase activity. From quantitation (Fig 4B, S5 Fig), we estimate that each of these three variants reduced kinase activity to approximately one-third of that of the wild-type protein. In principle, then, individuals who are homozygous for one of these alleles may have a lower level of cellular TNNI3K kinase activity than individuals who are heterozygous for a loss-of-function allele. Because the I686T variant in particular is so common across the human population, many individuals are homozygous for this allele (1.67% allele frequency and 0.06% homozygous frequency in ExAC). In mice, *Tnni3k* heterozygosity does not have a consequence on mononuclear CM content [20], so it is unlikely that heterozygosity of these hypomorphic variants would impact this parameter in humans. We do not yet have hypomorphic *Tnni3k* alleles in mice with which to define the threshold level of gene activity below which a measurable change in mononuclear CM frequency would result. Consequently, it is uncertain if homozygosity of the human hypomorphic alleles would impact CM composition.

The human variants may however be associated with heart disease. The rare human exon 4 splice donor site mutant that results in transcript nonsense-mediated decay, and the rare human T539A variant that is significantly hypomorphic in kinase activity (13% of wild-type activity; Fig 4), are both associated with adult conduction system disease and dilated cardiomyopathy as heterozygous alleles ([22, 23]; see also Introduction). Thus, the V510L, A666T, or I686T variants may predispose to these adult disease phenotypes, or may combine with other genetic or environmental conditions to result in these diseases.

### *Tnni3k* converges with oxidative stress to influence mononuclear CM frequency

**Fig 5:**
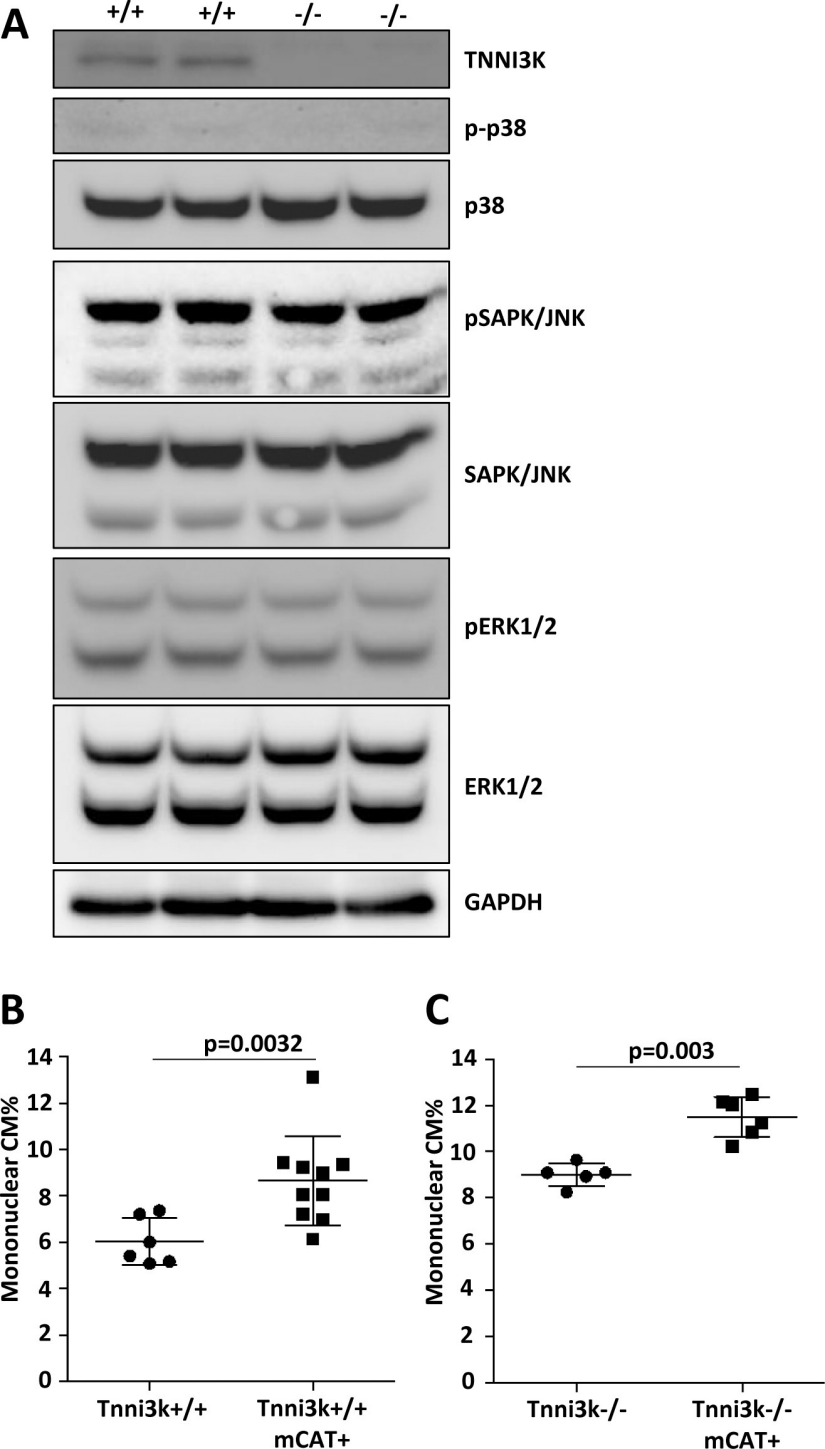
*Tnni3k* converges with oxidative stress. **A.** Western blot of ventricular protein from two wild-type and two *Tnni3k* null P3 mice showing phosphorylated (activated) and total protein for the three MAPK members p38, Jnk, and Erk. The very low level of phospho-p38 in P3 heart is compared against the normal level in adult heart in S6 Fig. **B-C.** Ventricular mononuclear CM% in adult hearts, comparing absence vs. presence of the mCAT transgene in a background that is wild-type for *Tnni3k* and heterozygous for *Nnt* (B), and comparing absence vs. presence of the mCAT transgene in a *Tnni3k* null and *Nnt* null background (C).

The critical time when mouse CMs become polyploid or remain mononuclear and diploid is midway through the first postnatal week [11]. There is little understanding of the mechanisms that mediate this transition, and virtually nothing known of how *Tnni3k* is involved in this process. As a MAP3K, a plausible mechanism to explain *Tnni3k* involvement is through phosphorylation of MAP2Ks (MEKs), which activate the MAP kinases Erk, Jnk, and p38. We tested for the level of phosphorylation of all three MAPKs in postnatal day P3 hearts (Fig 5A). We observed a prominent level of phosphorylated Erk and Jnk at this time point, but neither were affected by the absence of *Tnni3k*. The level of phospho-p38 was so low as to be barely detectable, but was not obviously different between wild-type and null P3 hearts. This level, however, was substantially lower than found in adult hearts (panel A of S6 Fig). Overall, this analysis provides no evidence for the involvement of MAPK signaling pathways as downstream mediators of Tnni3k kinase activity in influencing the neonatal transition of CMs to become polyploid.

In mice, a compelling case has been made that an increase in reactive oxygen species (ROS) associated with birth and postnatal life is a cause of cell cycle arrest [40], which is in principle equivalent to the onset of polyploidization. While excessive ROS (oxidative stress) is pathological and causes cell death, physiological levels of ROS are not damaging but rather serve as necessary intracellular signals. A primary ROS is superoxide, which is formed transiently in mitochondria and converted into the more stable peroxide. To experimentally modulate ROS level in mice, we used the mCAT transgenic line [41], which expresses catalase (which degrades peroxide) in mitochondria and so diminishes the amount of cellular ROS. The mCAT transgene was obtained on an inbred C57BL/6NJ background, which is a sister strain to C57BL/6J. We first tested to determine if this subtle difference in strain background had an impact on mononuclear CM frequency. Although there was a slight trend to higher mononuclear CM level in C57BL/6NJ mice, this difference was not statistically significant (panel B of S6 Fig). Nonetheless, because the main difference between the two strains is in the *Nnt* gene (functional in C57BL/6NJ, not functional in C57BL/6J [42]), in the following analyses we controlled for the *Nnt* status of all animals. By crossing the mCAT transgenic line with C57BL/6J mice to make an F1 generation (*Nnt* heterozygous), we first observed that mCAT expression alone resulted in an increase in mononuclear CM level (Fig 5B). This supports the interpretation that higher oxidative stress is associated with more CMs becoming polyploid, and conversely that lower oxidative stress associates with persistence of more mononuclear CMs. Crossing the mCAT transgene into the *Tnni3k* null background (now with the *Nnt* gene being null), we observed that mCAT expression still had the same effect of increasing mononuclear CM level, and that the effects of the two genetic manipulations were additive (Fig 5C). The nuclear ploidy of mononuclear CMs was not influenced by mCAT expression (S4 Fig, S5 Table). Thus, *Tnni3k* does not act as an obligate downstream mediator of oxidative stress, but rather converges with ROS level to influence the percentage of neonatal CMs that become polyploid or remain mononuclear diploid.

## Discussion

The *Tnni3k* gene is expressed almost exclusively in cardiomyocytes, a selectivity that is unique among the kinase family. Presumably, this relates to a gene function that was subjected to positive selection in evolution. Nonetheless, the *Tnni3k* gene can be mutated in mice without obvious deleterious consequence. As a result, a natural *Tnni3k* loss-of-function allele became widely distributed among inbred mouse strains, and gave the strongest association signal in our forward genetic screen for variants that affect the frequency of ventricular mononuclear CMs. Relatively little is known of the biological roles of this gene, and prior to our work it was unstudied in the context of the neonatal heart and the transition of neonatal cardiomyocytes to become polyploid.

By introducing a K489R point mutation into the *Tnni3k* gene in mice, resulting in a normally-expressed full-length kinase-dead protein, we show that Tnni3k functions as a kinase in the context of CM polyploidization. This was not a foregone conclusion, as the highly conserved amino-terminal ankyrin repeat domain constitutes over half of the protein length, and may have been the important functional domain in this process. The observation that the kinase-dead K489R mutant shows the same increase in mononuclear diploid CM content as alleles in which no Tnni3k protein is made demonstrates that the amino and carboxy terminal domains of the protein do not independently influence this frequency outside of their connection to kinase activity. They may also be required, but not independent of kinase function. The observation that mole-rat *Tnni3k* genes became mutated not only by acquiring point mutations in the kinase domain but also throughout the protein coding region is a further indication that the ankyrin repeat domain is unlikely to have a function independent of the protein’s kinase activity.

*Tnni3k* variants in mice influence the frequency of mononuclear diploid CMs. If the same pathways are active in the human heart, variants in the *TNNI3K* gene may similarly influence this trait in humans. Null *TNNI3K* alleles are present in the ExAC database, mostly as heterozygous alleles but with rare instances of humans that are homozygous null. If only limited to rare homozygous null individuals, the contribution of *TNNI3K* variants to variation in overall human heart composition would be of only incidental significance. More intriguing is the recognition that several common *TNNI3K* polymorphisms have approximately one-third the kinase activity of full-length protein, and that many individuals are homozygous for these hypomorphic alleles. These common human *TNNI3K* hypomorphic variants may therefore contribute to variation in mononuclear diploid CM level across a broad segment of the human population. As pointed out above, this assumes that the same pathways and the same role of Tnni3k in establishing mononuclear diploid CM level in the mouse heart are also similarly active in the human heart. A further caveat is that we only defined kinase activity of these variants using an in vitro assay and only using autophosphorylation as an experimental endpoint. The variants studied here may have more or less kinase activity in vivo on a true substrate protein compared to what was revealed by the in vitro assay. Furthermore, we cannot yet confirm what level of reduction of kinase activity would be needed to change mononuclear diploid CM level, as we do not have hypomorphic *Tnni3k* alleles in mice with which to model the effects of such variants.

In the neonatal heart, as CMs are induced to enter cell cycle, *Tnni3k* influences the percentage of these cells that successfully complete cell cycle with cytokinesis. This is consistent with an impact on the machinery of karyokinesis or cytokinesis, or on regulators of mitotic progression, rather than G1- or S-phase components. Even with the understanding that *Tnni3k* functions as a kinase in this context, a critical limitation is a lack of knowledge regarding its primary substrates and downstream pathways of activity. One mechanistic insight comes from an observation in adult heart ischemia-reperfusion injury where the presence of *Tnni3k* led to ROS overproduction and thereby to increased CM death [43]. This was shown to be mediated by p38, which is a known ROS sensor. Ischemia-reperfusion is a pathological situation of massive oxidative stress, and so observations made in this setting do not necessarily apply to the uninjured heart. Indeed, we examined MAPK components, including p38, and did not see an indication of relevance to the normal process by which neonatal CMs become polyploid. Even if the pathway of action of *Tnni3k* in neonatal CM polyploidy does not involve p38, convergence with ROS signaling is an appealing model. Under physiological conditions, lower ROS is associated with higher mononuclear diploid CM level; this was implied in a prior study showing the relation of oxidative stress to cell cycle interruption [40], and confirmed in our study with expression of mCAT to reduce ROS and increase mononuclear diploid CM content. Absence of *Tnni3k* has the same direction of effect as expression of mCAT. Conceptually, therefore, the normal function of *Tnni3k* may be to amplify ROS generation or signaling. The onset of CM polyploidy, which in mice occurs during the first postnatal week, is one specific time when this function of *Tnni3k* is manifest.

*Tnni3k* is expressed in CMs throughout life, so we expect it to have a continuing role in CM physiology, possibly also related to ROS signaling. *Tnni3k* function is maladaptive when the heart is challenged with extreme ROS levels as in the context of ischemia-reperfusion mentioned above, but given its evolutionary conservation, the gene is presumed to have a beneficial role under normal physiological circumstances, perhaps to augment responsiveness to metabolic alterations via ROS signaling. The apparent evolution of the *Tnni3k* gene in mole-rats to become nonfunctional (at least for protein coding) may be interpretable in this light. Mole-rats evolved to life in subterranean tunnels where oxygen levels are very low, but have a relatively pro-oxidant intracellular milieu and withstand normoxia without apparent ill effect, presumably by the activity of a variety of cytoprotective mechanisms [44, 45]. *Tnni3k* mediation of some feature of ROS response as we posit in other mammals may not have been needed or may even have been counterproductive for subterranean life. In human heart biology, though, diminishment or absence of this beneficial *TNNI3K* function may predispose to conduction system disease and dilated cardiomyopathy (as observed in the human pedigrees with these conditions), and perhaps more so if combined with other challenges that increase reactive oxygen generation. Clearly, it will be necessary to identify the kinase substrates and specific downstream pathways that might account for such effects to confirm this model.

One of the human disease-associated *TNNI3K* variants is the T539A point mutation that was included as a reference in our in vitro kinase analysis (Fig 4). This threonine lies in the “gatekeeper” position in the ATP binding pocket where it coordinates the adenine base of ATP. In all studied kinases, mutation of the gatekeeper residue to the smaller amino acids glycine or alanine enlarges the ATP binding pocket and reduce ATP affinity and thereby reduce kinase activity. As we measured, the T539A variant diminishes in vitro kinase activity to 13% of the wild-type protein. A second disease-associated *TNNI3K* variant is a splice donor mutation following exon 4 that was previously measured to cause nonsense-mediated decay, similar to the effect of several alleles explored in the present analysis. Importantly, both variants are disease-associated as heterozygous alleles. If these have hypomorphic or loss-of-function consequences, this implies that reduction of TNNI3K activity to below a threshold level even in the presence of one wild-type allele could cause disease manifestation. If so, the common hypomorphic variants described here (V510L, A666T, I686T), which based on the in vitro assay are predicted to result in 30–40% of kinase activity, may predispose to a similar spectrum of disease phenotypes. A recent hypothesis has suggested that disease-associated *TNNI3K* variants may instead have either hyperactive or dominant negative (sequestering) activity, rather than loss-of-function activity [25]. This is likely to be true for some disease-associated *TNNI3K* variants (e.g., E768K), although seems difficult to reconcile with nonsense-mediated decay of the exon 4 splice site mutation transcript and with the abundant biochemical information regarding the functional consequences of Ala substitution of the gatekeeper threonine as occurs with the T539A variant. If the common variants do contribute to disease as hypomorphic alleles, this may only be when combined with specific additional genetic or environmental perturbations. Possibly, families with conduction system disease and dilated cardiomyopathy share other genetic alterations or environmental conditions that combine with the heterozygous *TNNI3K* variants to cause disease.

A recent evaluation [46] concluded that the acquisition of endothermy (generating body heat by metabolism, commonly described as warm-blooded biology) in animal evolution was associated with thyroid hormone-dependent metabolism, causing increased levels of CM polyploidy and thereby loss of adult heart regenerative capacity. Naked mole-rats are an interesting test of this model, in that they are one of the few mammalian species that are poikilothermic (do not regulate their body temperature), and have a low basal metabolism and a low level of thyroid hormone [47]. Our analysis indicates that the level of mononuclear diploid CMs in *H*. *glaber* is on the higher side of the range seen in inbred mouse strains but is certainly not remarkable. This species would appear to contradict the model that thyroid hormone and endothermy are a primary explanation for CM polyploidy. *H*. *glaber* may be an exception to this rule, or the model may not fully account for this process. *H*. *glaber* presumably evolved to poikilothermia long after a high level of CM polyploidy evolved in endothermic vertebrates, and may have retained additional mechanisms that drive CM polyploidy even as they lost warm-blooded metabolism.

## Methods

### Animals

All mouse experiments were performed on age- and sex-matched animals (8–10 weeks of age), with an equal ratio of male to female mice. Naked mole rats were provided by T.J.P. from his colony at the University of Illinois at Chicago and sent as live animals to USC where hearts were isolated for analysis.

### Ethics Statement

Animal research was reviewed and approved by the IACUC committees of the Univ. of Southern California (#10173) and of the Medical Univ. of South Carolina (201800642). Animals were euthanized by isoflurane anesthesia followed by cervical dislocation and removal of hearts.

### Derivation of new mouse *Tnni3k* alleles

The K489R allele was derived by the VCU Massey Cancer Center Transgenic/Knockout Mouse Core. The protospacer/PAM target sequence 5’-AGATGAATTGCATCGTCAGCTGG-3’, which is in the 5’ side of *Tnni3k* intron 15, was used to generate an Alt-R crRNA, which was annealed to the Alt-R tracrRNA (both from Integrated DNA Technologies, IDT) to generate the functional guide RNA. Homology-directed repair at the cleavage site utilized a 200-base single-stranded oligodeoxynucleotide (ssODN) identical to the WT locus non-coding strand spanning from intron 14 to exon 16, with the exception of three altered bases: an A-to-G substitution to create the lysine-to-arginine mutation at codon 489, and two G-to-T substitutions to eliminate the Cas9 PAM sequence (to prevent retargeting) and to create a DraI site for screening purposes. A mix containing 1.2 μM Cas9 protein (IDT Alt-R S.p. HiFi Cas9 Nuclease 3NLS), 6 μM annealed cr/tracrRNA (preincubated with the Cas9 protein at room temperature for 10 min), and 300 ng/μl ssODN (IDT) in Opti-MEM medium (Thermo Fisher Scientific) was electroporated into fertilized C57BL/6J mouse eggs using a NEPA 21 electroporator (NEPA GENE Co. Ltd., Chiba, Japan) and CUY501P1-1.5 electrode as follows: 5 μl containing ~30 embryos at a time received 4 “poring pulses” (40V, 3.0 msec, 50 msec intervals, 10% voltage decay rate, polarity +) followed by 5 “transfer pulses” (7V, 50 msec, 50 msec intervals, 40% voltage decay rate, polarity +/- alternating.) The impedance of the solution on the electrode was maintained at ~0.20–0.22. Following electroporation, the eggs were placed back into KSOM medium (Specialty Media), and subsequently implanted into the oviducts of pseudopregnant ICR females (~15 embryos per recipient). At weaning, DNA was purified from a 5 mm tail snip from each pup. The PCR genotyping protocol employed primers flanking the targeted region, (forward: 5’- AAGCCCAGACCATGTGTGCTAAGG-3’ and reverse: 5’- CAGTAGGGTCTCTGACTGGAAGTC-3’), yielding a 775-bp product for both the WT and KI alleles. Subsequent digestion of the product with DraI yielded 428bp and 347bp fragments for the KI allele, while the WT allele remained uncut. The Δ4 and Δ8 alleles were generated at the Children’s Hospital of Los Angeles Saban Research Institute Transgenic/Knockout Mouse Core, using the same IDT crRNA and tracRNA procedures described above with a protospacer/PAM target sequence of 5’-GTGACAATGGCAAACTGGCTGGG-3’, which is located within exon 16. The annealed crRNA-tracrRNA complex was diluted in injection buffer (1 mM Tris-HCl, pH 7.5, 0.1 mM EDTA) to 40 ng/μl and mixed with equal volume of Alt-R S.p. HiFi Cas9 nuclease 3NLS (at a concentration of 40 ng/μl in injection buffer). The mixture of crRNA-tracrRNA and Cas9 nuclease were incubated at room temperature for 15 min to form crRNA-tracrRNA-Cas9 protein ribonucleoprotein complex used in pronuclear injection. The final concentration of crRNA-tracrRNA and Cas9 ribonucleoprotein were both 20ng/μl. Pronuclear injection of E0.5 embryos was performed according to standard procedures; after injection, viable embryos were transferred to pseudopregnant CD1 female mice. For genotyping, primers flanking the targeted region (forward: 5’-AAAACACCCCGTGATGTTTTATT-3’ and reverse: 5’- CAGATGAATTGCATCGTCAGCT-3’) yielded a 942bp (wild-type allele; slightly smaller for deletion alleles) product that was gel purified and then sequencing using the primer 5’-TAGATACCGAGCCAACACC-3’.

### Mouse allele and genotype designations

The wild-type *Tnni3k* allele in C57BL/6J mice is indicated as the + allele. An engineered loss-of-function *Tnni3k* null allele described previously [20] is designated as the - allele. The alleles newly created in this study are represented by Δ4, Δ8, and K489R. Unless otherwise noted, alleles were combined such that all animals carried one null allele (e.g., +/-, Δ4/-, Δ8/-, K489R/-, and -/-). The mCAT transgenic allele was obtained from The Jackson Laboratory (JAX016197) and in all cases was kept as a hemizygous allele.

### Single-cell ventricular cardiomyocyte suspensions

Hearts were digested with 1mg/mL collagenase type II via Langendorff retroaortic perfusion. After digestion, atria and valves were removed and ventricular tissue alone was triturated in Kruftbrühe (KB) solution (70mM potassium aspartate, 40mM KCl, 15mM KH_2_PO_4_, 10mM glucose, 10mM taurine, 0.5mM EGTA, 10mM sodium pyruvate, 10mM HEPES, 5mM BDM, 0.5% BSA), filtered through a 250-μm nylon mesh, stained with LiveDead Fixable (ThermoFisher, L10120) for 20 min at room temperature and then fixed in 2% PFA at room temperature for 15 min. Fixed ventricular cell suspensions were stained for cTnT (1:1,000, Abcam ab8295) overnight at 4°C followed by goat anti-mouse secondary (1:500, ThermoFisher A11001) and DAPI. Cell suspensions were then pipetted across a slide and coverslipped. Cardiomyocyte nucleation was quantified on an Olympus BX41 fluorescence microscope with a 20x objective. Only live cardiomyocytes were counted for mono-, bi-, tri- and tetranucleation; at least 600 cells were counted per heart. An unpaired, two-tailed Student t-test was used to assess statistical significance when only two groups were compared.

### Cardiomyocyte ploidy analysis

Cardiomyocyte suspensions were spread on slides and stained for mouse anti-Myl2 (1:250) and anti-CD31 (1:250) with an Alexa Fluor secondary (1:250, ThermoFisher A11001) and DAPI using standard procedures. Cells were coverslipped with ProLong Gold antifade reagent (Invitrogen). Slides were scanned by fluorescence microscopy. IMARIS software was used to quantify cardiomyocyte ploidy. Briefly, nuclei were identified and outlined with a standard threshold requirement for all samples. A histogram of each nucleus was generated and total fluorescence was calculated as area under the curve, thereby accounting for both size of the nucleus and fluorescence intensity. The median value of DAPI fluorescence intensity of CD31+ endothelial cell nuclei was used as a diploid nucleus standard.

### Reverse transcription polymerase chain reaction (RT-PCR)

RNA was extracted from heart ventricle using Quick-RNA Mini Prep Kit (Zymo Research). Equal amounts of RNA between samples were used to synthesize cDNA using M-MLV reverse transcriptase (Invitrogen). Primer sequences used to amplify *Tnni3k* cDNA to detect its relative abundance across samples were: 5′-CAGCACAGGAGGAAAGCAGA-3′ and 5′-GCAGGTCATCTTCCAGCCTT-3′. Gapdh was used as an internal control.

### Plasmids and molecular cloning

Mouse cDNA was synthesized using SuperScript III First-Strand Synthesis System (Invitrogen). The mouse *Tnni3k* gene was amplified from C57BL/6J mouse cDNA by PCR with Q5 High-Fidelity DNA Polymerase (New England Biolabs) and the primers forward: 5’-GAGAGAAGATCTGCCACCATGGGGAATTACAAATCCAGACCG-3’ and reverse: 5’-GAGAGACTCGAGTTACTTGTACAGCTCGTCCATGCCG-3’. The naked mole rat *Tnni3k* gene was amplified from *H*. *glaber* cDNA by PCR with primers forward: 5’-GAGAGAGGATCCGCCACCATGGGAAATTATAAATCTAGACCAACACA-3’ and reverse: 5’- GAGAGACTCGAGTCAACAGTGTTTTATAGCCACTATTTTATTTCT-3’. Amplified fragments were cloned into the pcDNA3 vector (Addgene) with an N-terminal Flag tag. All *Tnni3k* point mutant expression plasmids (K489R, T538A, V509L, S590T, T636M, A665T, I685T), and all truncation mutants (Δ4, Δ8, 492*) were constructed from the wild-type complete open reading frame pcDNA3-Flag-Tnni3k plasmid.

### Western blotting

Heart ventricular tissue was snap frozen in liquid nitrogen then homogenized with an OMNI TH homogenizer in lysis buffer (50mM Tris-HCl pH7.5, 150mM NaCl, 0.1% NP-40) on ice. Heart lysates were centrifuged at 14,000 RPM for 20 min at 4°C to remove insoluble material. Lysis buffer contained 1x Complete protease inhibitor mixture (Roche), and 1x PhosStop phosphatase inhibitor (Roche). Immunoblotting was performed by standard protocols with 50μg heart lysate using anti-Tnni3k (1:1,000, Invitrogen PA5-21989), anti-GAPDH (1:1,000, GeneTex GT239), anti-p38 (1:1,000, Cell Signaling Technology, CST9212), anti-phospho-p38 (1:1,000, Cell Signaling Technology, CST4511), anti-ERK1/2 (1:1,000, Cell Signaling Technology, CST4695), anti-phospho-ERK1/2 (1:1,000, Cell Signaling Technology, CST9101), anti-SAPK/JNK (1:1,000, Cell Signaling Technology, CST9252), anti-phospho-SAPK/JNK (1:1,000, Cell Signaling Technology, CST4668) and HRP-coupled secondary (1:10,000, Jackson ImmunoResearch sc2040) antibodies. 20μg HEK-293T cell lysate or 1μl of beads with immunoprecipitated TNNI3K were loaded and blotted in the same manner, then using anti-phospho-tyrosine (1:1,000, Cell Signaling Technology, CST9411), anti-phospho-serine (1:1,000, Millpore Sigma, AB1603), anti-Flag (1:1,000, Invitrogen, MA1-91878), or anti-β-catenin (Ser33/37/Thr41) (1:1,000, Cell Signaling Technology, CST8814). Quantification of Western blotting signal was done using ImageJ.

### Tissue culture, transfection and immunoprecipitation

HEK293T cells were grown using standard conditions in DMEM (Gibco) containing 10% (vol/vol) FBS (Equitech-Bio, Inc.), 1x sodium pyruvate (Gibco), 1x MEM NEAA (Gibco), and 1x penicillin-streptomycin (Gibco). Cells were transfected with Lipofectamine 3000 (Invitrogen) at 60% confluence for 24h, and harvested at 90–100% confluence. Cell lysis was performed in lysis buffer (50mM Tris pH7.5, 150mM NaCl, 0.1% NP-40, 1x Roche Complete protease inhibitor mixture, and 1x PhosStop phosphatase inhibitor (Roche)). MG132 (Sigma-Aldrich) was applied 24h after transfection for 8h before harvest.

### In vitro kinase assays

Immunoprecipitation of protein was performed from 1mg HEK293T cell lysate per sample, using 10μL of anti-Flag magnetic beads (Sigma) at 4°C overnight. For use in in vitro kinase assays and Western blotting, beads were washed five times in 500μL lysis buffer, and resuspended in 10μL lysis buffer. 1μL of immunoprecipitated flag-tagged TNNI3K protein on beads was used for in vitro kinase assay, conducted in 50mM Tris pH7.5, 150mM NaCl, 10mM MgCl_2_, 20 μM ATP, 2mM DTT. The reactions were incubated at 30°C with gentle agitation for 30 min. Reactions were quenched by adding gel loading buffer and heating to 95°C before SDS/PAGE separation.

## Supporting information

S1 Fig Sequence of the Δ4 and Δ8 alleles. Sequence traces of genomic DNA from heterozygous Δ4/+ (top) and Δ8/+ (bottom) mice, illustrating the location where the sequences of the wild-type and deletion mutant alleles diverge. The deleted bases correspond to positions 154939648–651 (Δ4) and 154939641–648 (Δ8) of NC_000069.6. Both alleles result in frameshifts that cause downstream sequences to become termination codons (in red).
(PDF)

S2 Fig The *Tnni3k* gene in *H. glaber* and *F. damarensis* mole-rats. **A.** Alignment of portions of mouse exons 16, 20, and 21 with genomic DNA from the two mole-rat species (see S2 Table and S3 Table for coordinates of the mole-rat exon sequences), emphasizing the premature stop codons in exons 16 and 21 and the gap in exon 20 in *H*. *glaber* (in the center line), whereas F. damaraensis conserves the open reading frame in these regions. **B.** Sequence traces from *H*. *glaber* genomic DNA of the portions of exons 20 and 21 shown in panel A. **C.** Putative mRNA and translated reading frame as deduced from *H*. *glaber* genomic DNA, beginning with the ATG codon in exon 1 and ending with the TGA codon in exon 25. The premature stop codon in exon 16 is underlined and in red type. The putative translated product begins with the ATG codon in exon 1 and ends at the premature stop codon in exon 16. **D.** Alignment of the putative translated *H*. *glaber* (Hgla) Tnni3k protein with that of mouse (Mmu). **E.** Sequence of the *Tnni3k* cDNA as obtained experimentally from *H*. *glaber* ventricular mRNA, beginning with the ATG codon in exon 1 and ending with the premature stop codon in exon 16. The first in-frame stop codon is underlined and in red type. The translated product is shown below. **F.** Alignment of portions of mouse exons 5 and 15 with genomic DNA from the two mole-rat species, here with *F*. *damarensis* in the center line and organized to show the gaps and premature stop codons in both exons, whereas *H*. *glaber* conserves the open reading frame in these regions. Sequence traces of *F*. *damarensis* for these exon regions are shown below.
(PDF)

S3 Fig Construction of the K489R kinase dead *Tnni3k* allele in mice. **A.** Diagram of the wild-type allele and the conversion of the AAA codon encoding K489 to AGA (Arg). An intronic TGGAAA sequence was converted at the same time to the DraI restriction site TTTAAA. **B.** Sequence trace of a K489R/+ mouse, illustrating the changes introduced into the gene as in panel A. **C.** DraI restriction digest of PCR-amplified genomic DNA from mice of the indicated genotypes.
(PDF)

S4 Fig Measurement of nuclear ploidy. **A.** Graphical representation of nuclear ploidy in mononuclear and binuclear CMs from mice of the indicated genotypes. The first three columns of the Mononuclear panel are duplicated from Fig 3C for easier comparison to the remainder of the figure. **B.** Primary data evaluating mononucleated and binucleated CM populations for nuclear DAPI fluorescence in ventricle cell preparations from mice of the indicated *Tnni3k* genotypes. Each dot represents one nucleus. Numbers above plots indicate animal identifiers, and compiled numerical data are shown in S5 Table.
(PDF)

S5 Fig Quantitation of kinase activity of Tnni3k variants in an in vitro kinase reaction. 293 cells were transfected with plasmids to express full-length wild-type mouse Tnni3k or human variants introduced into the mouse sequence (numbering based on the mouse protein, which is one less than the human protein). Within a group of three plasmids, each was transfected in the same experiment and cell lysates prepared and used in an in vitro kinase reaction at the indicated time points; all samples were run on gels and blotted at the same time and then probed with antibody and visualized together. Each variant was assayed twice, and all were assayed with the wild-type construct as an internal reference. **B.** Quantitation of the normalized signals from the blots shown in panel A. Each experiment was quantitated individually because blotting or antibody conditions and exposure times may have varied between individual experiments.
(PDF)

S6 Fig Tnni3k signaling pathways. **A.** Comparison of phospho-p38 signal in the same four P3 samples shown in Fig 5A, now also with two wild-type adult heart samples included on the same blot. **B.** Ventricular mononuclear CM% in adult hearts, comparing C57BL/6J vs. C57BL/6NJ mice.
(PDF)

S1 Table *Tnni3k* homologs in other species. Accession numbers for *Tnni3k* or close homologs in human, mouse, sea urchin (*S*. *purpuratus*), golden apple snail (*P*. *canaliculate*) and Cailifornia two spot octopus (*O*. *bimaculoides*) are provided.
(DOCX)

S2 Table Candidate assembly of *Tnni3k* gene in *Heterocephalus glaber* from NW_004624742.1 by homology to mouse gene. (DOCX)

S3 Table Candidate assembly of *Tnni3k* gene in *F. damarensis* from NW_011046421.1 by homology to mouse gene. (DOCX)

S4 Table Relatively common human *TNNI3K* kinase-domain variants (from ExAC). (DOCX)

S5 Table Compilation of diploid nuclei percentages of mononucleated (Mono) and binucleated (Bi) cardiomyocytes in this study. These data correspond to Figs. 2D, 3B, 5B and 5C.
(DOCX)
