# Supplementary figures and images for: Tnni3k alleles influence ventricular mononuclear diploid cardiomyocyte frequency
Source: PLoS Genet. 2019 Oct 7;15(10):e1008354. doi: 10.1371/journal.pgen.1008354 (PMC6797218; doi:10.1371/journal.pgen.1008354)

# Figure S1

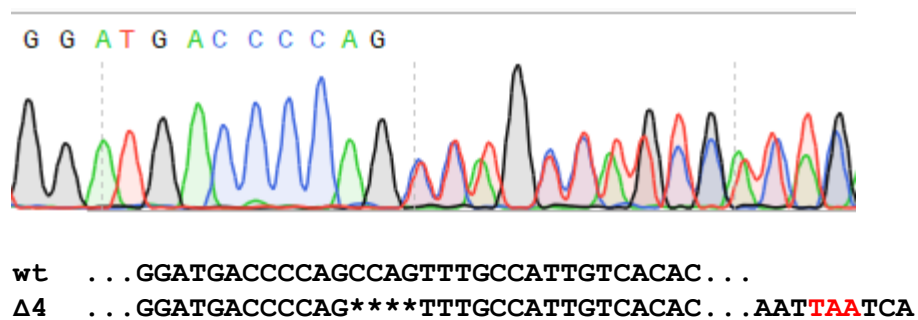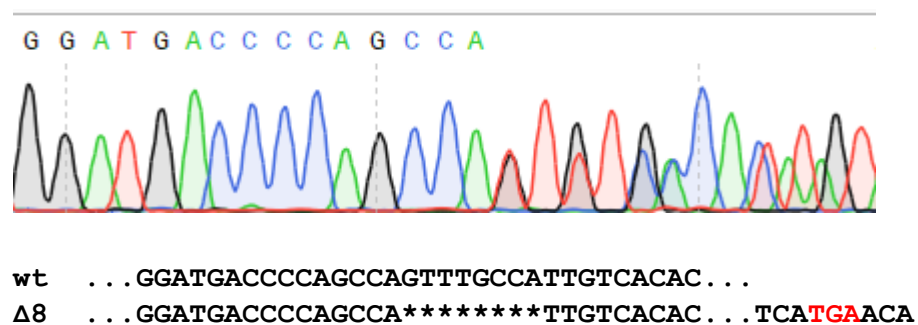

Supplement: S1 Fig — Sequence traces of genomic DNA from heterozygous Δ4/+ (top) and Δ8/+ (bottom) mice, illustrating the location where the sequences of the wild-type and deletion mutant alleles diverge. The deleted bases correspond to positions 154939648–651 (Δ4) and 154939641–648 (Δ8) of NC_000069.6. Both alleles result in frameshifts that cause downstream sequences to become termination codons (in red). (PDF) [file pgen.1008354.s001.pdf]

# Figure S3

**A**

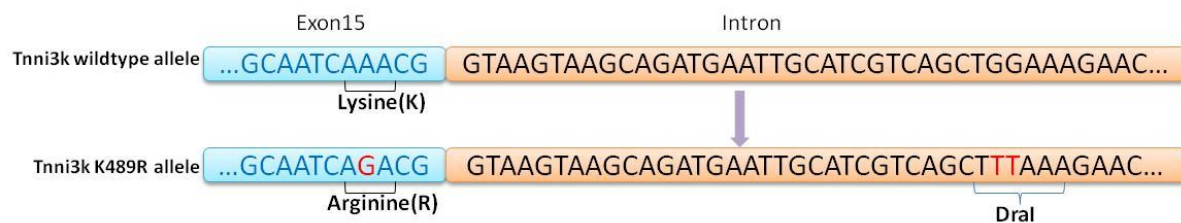

**B**

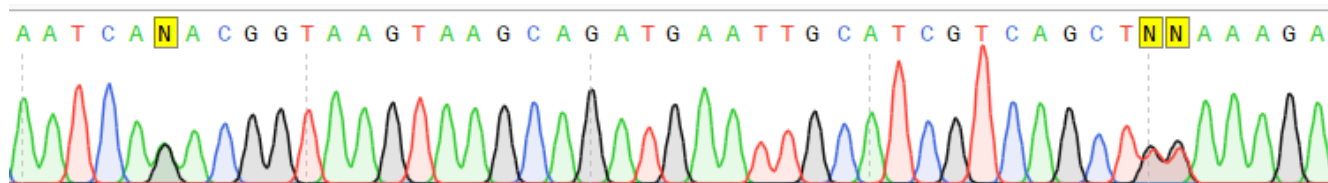

**C**

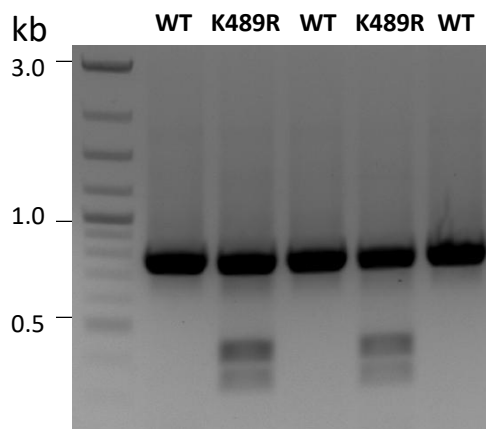

Supplement: S3 Fig — A. Diagram of the wild-type allele and the conversion of the AAA codon encoding K489 to AGA (Arg). An intronic TGGAAA sequence was converted at the same time to the DraI restriction site TTTAAA. B. Sequence trace of a K489R/+ mouse, illustrating the changes introduced into the gene as in panel A. C. DraI restriction digest of PCR-amplified genomic DNA from mice of the indicated genotypes. (PDF) [file pgen.1008354.s003.pdf]

Figure S4

A

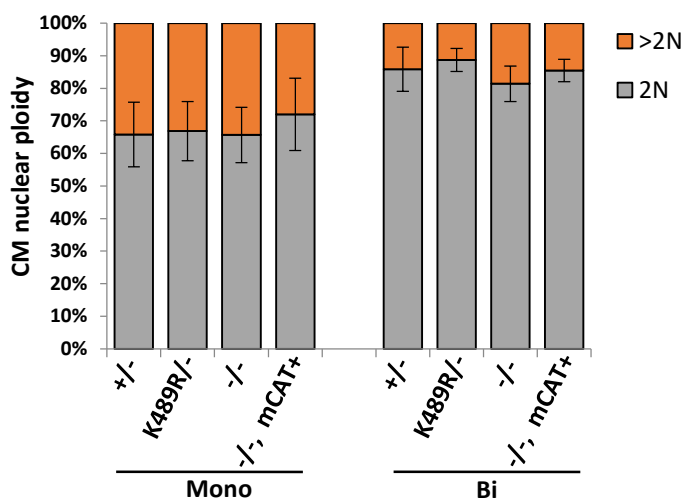

# Figure S4 continued

**B**

*Tnni3k* +/-

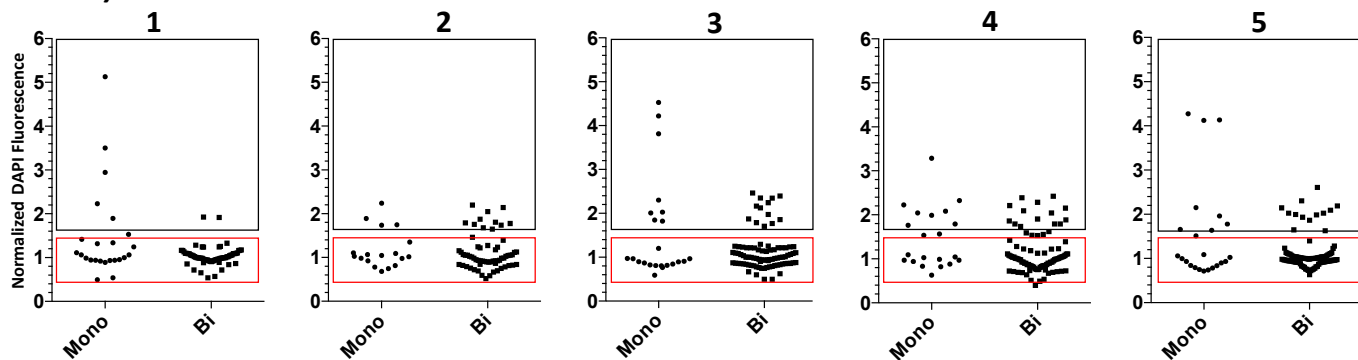

*Tnni3k* K489R/-

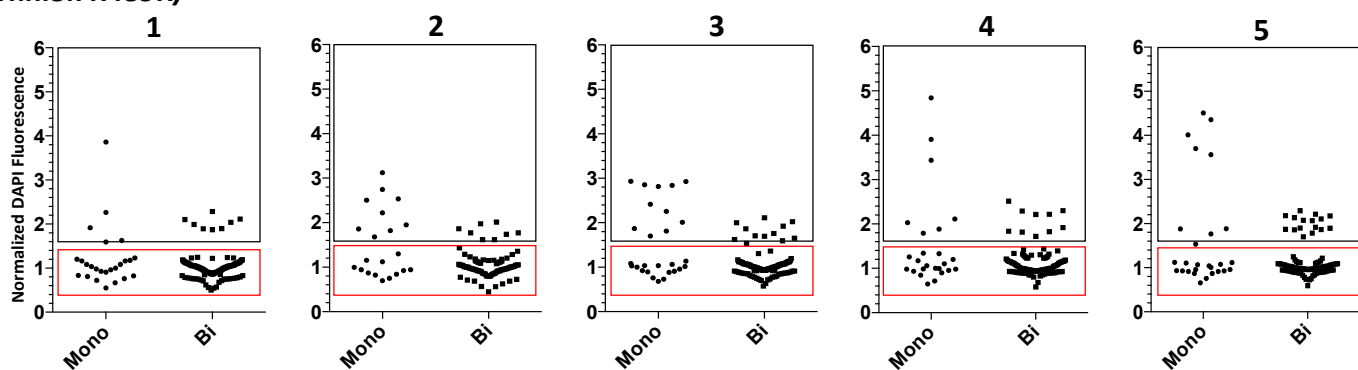

*Tnni3k* -/-

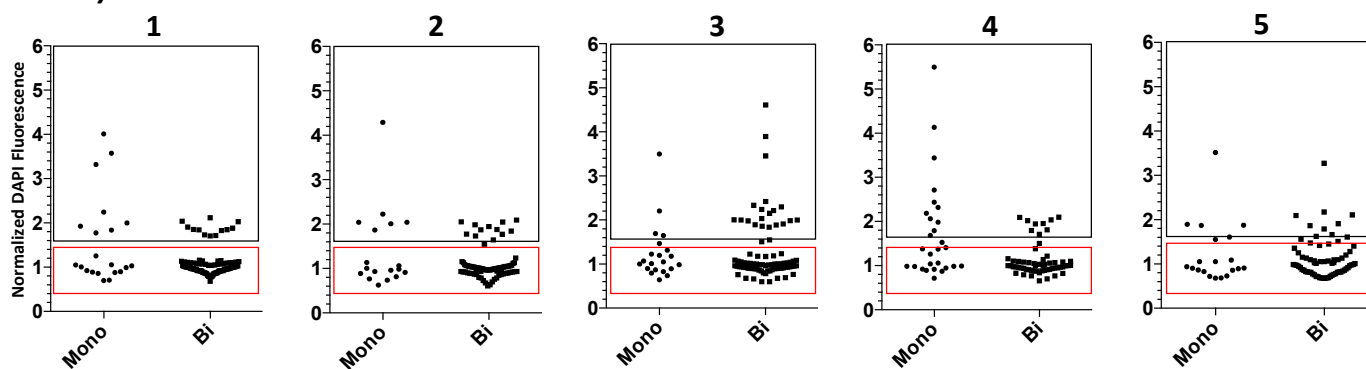

*Tnni3k* -/-, mCAT+

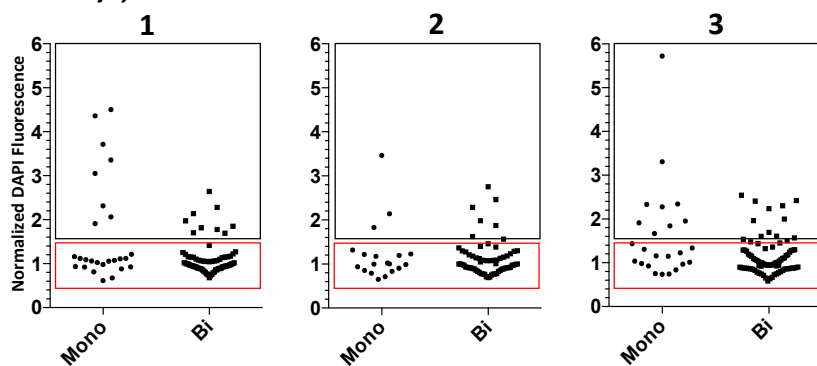

Supplement: S4 Fig — A. Graphical representation of nuclear ploidy in mononuclear and binuclear CMs from mice of the indicated genotypes. The first three columns of the Mononuclear panel are duplicated from Fig 3C for easier comparison to the remainder of the figure. B. Primary data evaluating mononucleated and binucleated CM populations for nuclear DAPI fluorescence in ventricle cell preparations from mice of the indicated Tnni3k genotypes. Each dot represents one nucleus. Numbers above plots indicate animal identifiers, and compiled numerical data are shown in S5 Table. (PDF) [file pgen.1008354.s004.pdf]

Figure S5

A

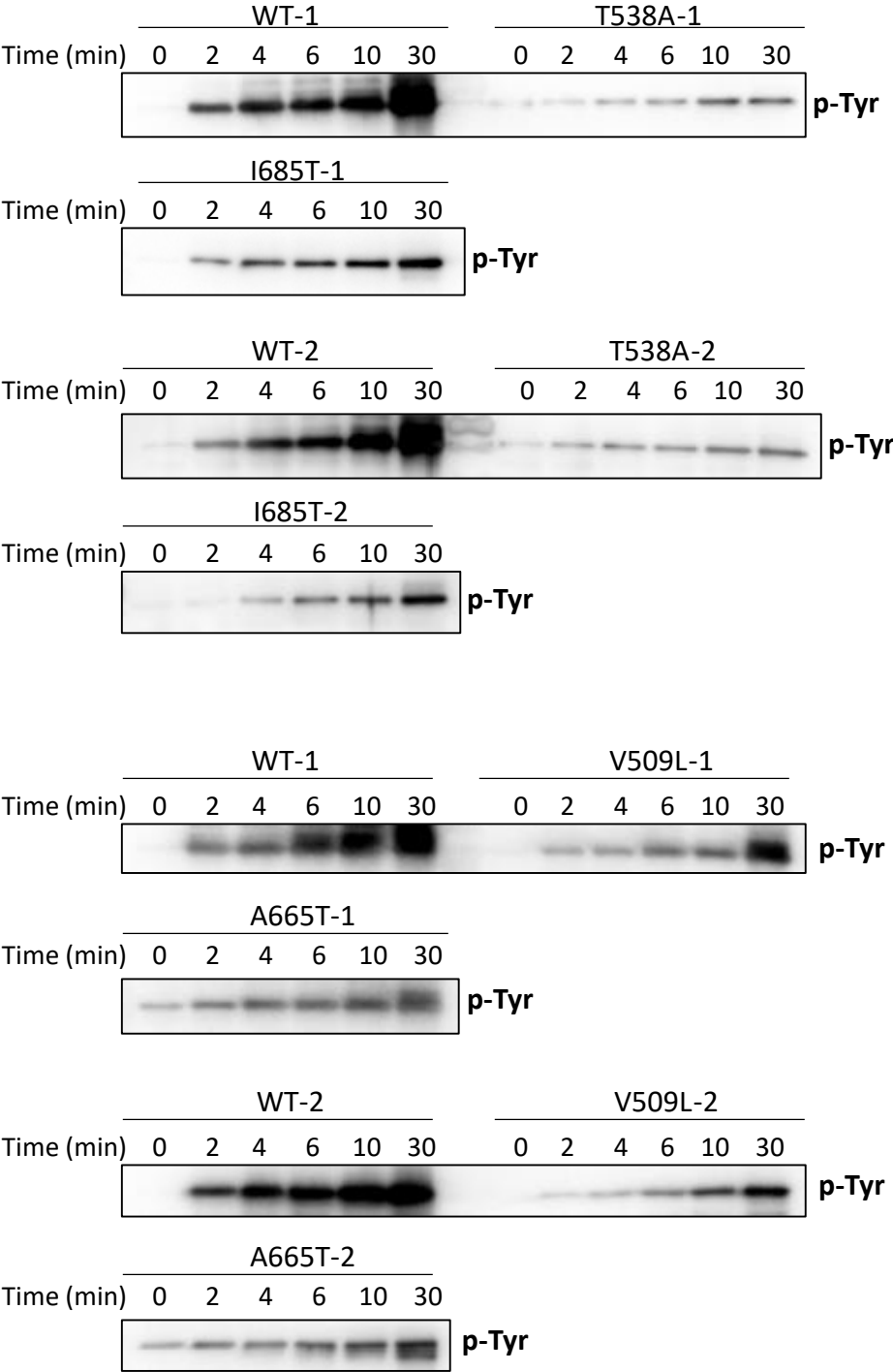

Figure S5 continued

B

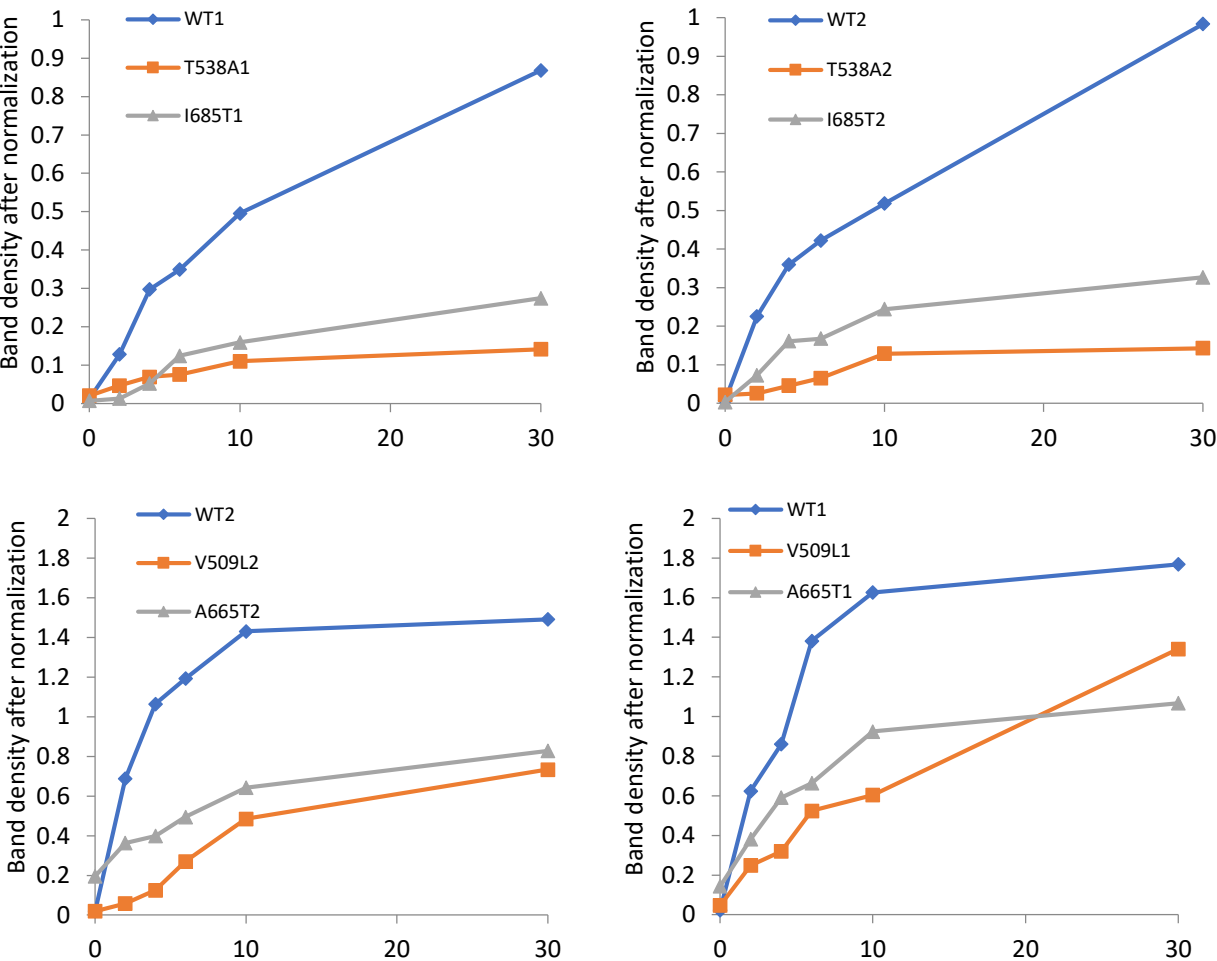

Supplement: S5 Fig — 293 cells were transfected with plasmids to express full-length wild-type mouse Tnni3k or human variants introduced into the mouse sequence (numbering based on the mouse protein, which is one less than the human protein). Within a group of three plasmids, each was transfected in the same experiment and cell lysates prepared and used in an in vitro kinase reaction at the indicated time points; all samples were run on gels and blotted at the same time and then probed with antibody and visualized together. Each variant was assayed twice, and all were assayed with the wild-type construct as an internal reference. B. Quantitation of the normalized signals from the blots shown in panel A. Each experiment was quantitated individually because blotting or antibody conditions and exposure times may have varied between individual experiments. (PDF) [file pgen.1008354.s005.pdf]

Figure S6

A

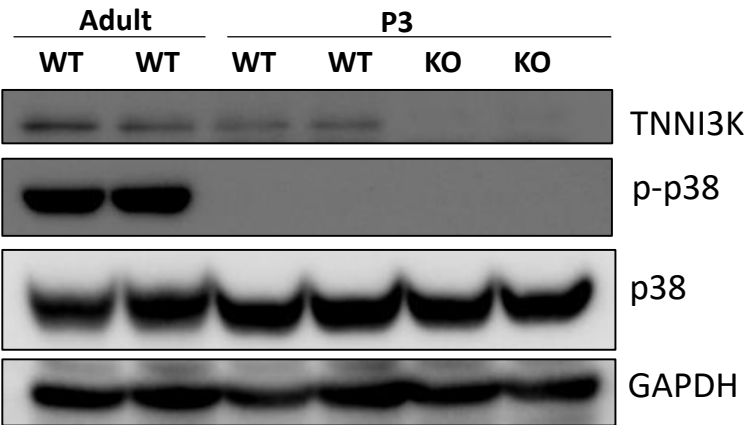

B

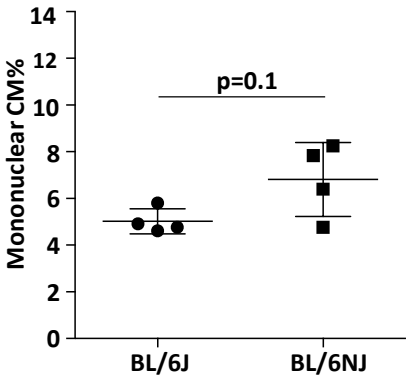

Supplement: S6 Fig — A. Comparison of phospho-p38 signal in the same four P3 samples shown in Fig 5A, now also with two wild-type adult heart samples included on the same blot. B. Ventricular mononuclear CM% in adult hearts, comparing C57BL/6J vs. C57BL/6NJ mice. (PDF) [file pgen.1008354.s006.pdf]
