# Supplementary material for: Tnni3k alleles influence ventricular mononuclear diploid cardiomyocyte frequency
Source: PLoS Genet. 2019 Oct 7;15(10):e1008354. doi: 10.1371/journal.pgen.1008354 (PMC6797218; doi:10.1371/journal.pgen.1008354)

Figure S2

A

```
Exon 16
            Y  R  A  N  T  Y  C  S
Mmu ...agATACCGAGCCAACACCTACTGCTCC...
Hgla...agCTGTTGAGCCAACACCTATTGCTCC...
Fdam...agTTATCGAGCCAACACCTACTGCTCC...
            Y  R  A  N  T  Y  C  S

Exon 20
           N  L  R  W  M  A  P  E  V  F  T
Mmu ...agAACCTGCGCTGGATGGCCCCCGAGGTGTTCACA...
Hgla...agAACCTGCGCTGGATGGCCCGGG-GGTGTTCACG
Fdam...agAACCTGGGCTGGATGGCGCCGGAGGCGTTCACA...
           N  L  G  W  M  A  P  E  A  F  T

Exon 21
        I  S  S  L  L  M  R  G  W  N  A  C
Mmu ...ATCTCATCTCTGCTGATGCGGGGCTGGAATGCATGT...
Hgla...ATATCCTCTCTGCTGATGTGAGGTTGGAATGCGTGT...
Fdam...ATATCTTCTCTGCTTATGCAATGTTGGAATGTATGT...
        I  S  S  L  L  M  Q  C  W  N  V  C
```

B

H. glaber exon 20

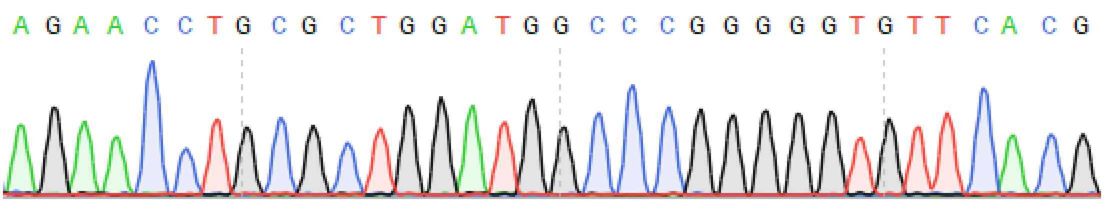

H. glaber exon 21

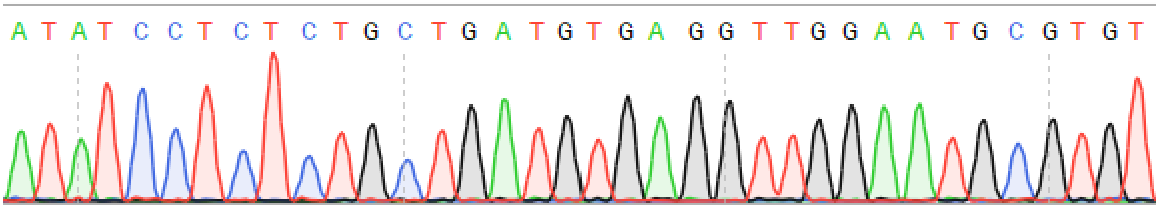

Figure S2 continued

C

ATGGGAAATTATAAATCTAGACCAACACAAACTTGTACTGATGAGTGGAAGAAGAAAGTCAATGAATCTT
ATGTTATTACAATAGAAAGATCAGAAGATGACCCGAGGATCAAGGAAAAAGAATTTACAGAGATGAGACA
TATTTTTGGCTCTGATGAAGCCTTCAGTAAAGTCAACCTAAATTACCGCACTGAAAGTGGACTATCTCTA
CTGCATTTATGTTGCATTTGTGGAGGCAACCAGTCTCATATTCGAGCCCTTATGTTGAAAGGACTCTGCC
CATCTCTACTGACAAGAAATGGATTCACAGCGTTGCACCTAGCAGTTTATAAGGACAGTGCAGAACTGAT
CACTTCCTTCCTCCACAGTGGGGGTGATGTGCAGCAGGTGGGGTTCGGTGGCCTCACTGCACTCCACATT
GCTGTCATCGCTGGTCACCTGGAGGCCCCTGATGTGTTACTCCAACACGGAGCTAATGTCAATGTTCAAG
ATGCTGTTTTTTTCACTCCATTGCACATTACAGTGTACTATGGGCATGAGCAGGTAACCCATCTCCTTTT
GAAATTTGGTGCTGATGTAAATGTAAGTGGTGAAGTTGGGGATAGGCCTCTCCATCTGGCATCTGCAAAG
GGATTCTTCAACATTGCAAAACTCCTGATGGAAGATGGGAATAAAGCAGATGTGAATGCTCAGGATAATG
AAGACCATGACCCACTGCATTTCTGTTCTCATTTTGGCCATCACGAGATAATGAAGTATCTGCTCCAAAG
TGACTCGGAAGTTCAGCCTCATGTTGTTAATATCTATGAAGACACTCCTTTGCACTTGGCATGCTACAAT
GGCAAATTTGAAGTTGCTAAGGAGATCATTCAAATAACTGGAACAGAAAGTTTGACTAAGGAAAACATCT
TTAGTGAGACAGCTTTTCACAGTGCTTGTACCTATGGCAAGAGCATTGATCTGGTTAAATTTCTTCTTGA
TCAGAATGTGGTAAACATCAGTCACCAAGGAAGAGATGGGCACACAGGATTACACTCTGCTTGCTACCAT
GGTCACATTCACCTGGTTCAGTTCTTACTGGACAATGGGGCTGATATGAATCTAGTTGCTTGTGATCCCA
GCAGGTCTAGTGGTGAAAAAAATGAGCAGACATGTTTGATGTGGGCTTATGAAAAAGGACAGGATGCCAT
TGTCACACTTCTGAAGCATTATAAGAGACCCCAGGATGAACTGCCCTGTAATGAATATTCTCGTCCTGGA
GGAGATGGCTCCTATGTGTCTGTTCCATCCCCTTTGGGGAAGATTAAAAACATAACAAAAGAGAAGGTGG
ATGTTCTCCTCCTGAGGGCCAGATTGCCTTCACATTTCCATCTTCAGCTCTCGGAAATTGAGTTCCATGA
GATTATCGGCTCAGGTTCTTTTGGGAAAGTATACAAAGGACGATGCAGAAATAAAATAGTGGCTATAAAA
CACTGTTGAGCCAACACCTATTGCTCCAAATCAGATATGGATATGTTTTGCCGAGAGGTGTCCATTCTCT
GCCAGCTCAATCATCCCTGTGTCATTCAGTTTGTGGGCACTTGCTTAAATGATCCCAGCCAGTTTGCCAT
TGTCACCCAGTACATCTCAGGAGGTTCTCTGTTCTCCCTCCTTCATGAGCAGAAAAGGGCCCTTGATTTG
GAGTCTAAGTTAATTATTGCAGTAGATATTGCCAAAGGCATGGAGTACCTTCACCACCTGACCCAGCCAA
TTATACACCGTGACTTGAACAGTCACAATATTCTTCTCTATGAGGACGGGCATGCTGTGGTGGCCGATTT
TGGAGAATCAAGATTTCTACAATCTCTGGATGAAGACAACATGACAAAGCAACCTGGGAACCTGCGCTGG
ATGGCCCGGGGGTGTTCACGCAGTGCGCCCGCTACACCACTAAGGCCGAAGTCTTCAGCTGCTCGCTGTG
TCTGGGGGAGCTGCTGACTGGTGAGATTCCCTTCGCTCATCTCAAGCAGTGGCAGCGGCAGCAGACATGG
CCTCCCACCACATCAGGCATCCCATTGGATATTCCATTCCCAAGCCCATATCCTCTCTGCTGATGTGAGG
TTGGAATGCGTGTCCTGAAGGAAGACCTGAGTTTTCCAAAGTTGTTACCAAGTTAGAAGAATGTCTCTGC
AACATTGAGCTGATGTCTCCGGCATCGAGTAACAGCAGTGGGTCTCTTTCACCTTCCTCTTCTTCTGATT
GCCTGGCAAGCCAGGGAGCCCCTGGCCAGAGCACGTGGCAGCTTTACGGAGCCGTTTTGAGTTGGAGTGT
GCTCTCAACGCAAGGTCCTATGCTGCCTGGCCCCAAAGCACTGGACCAAGTTCTCCTCAGGGGCTGTCTT
TGGAGGAGATGTGCAGGAGTCTCCAGTGTTCCACCATCGACAAATACGGCTACATGTCTGATCCCCTGAG
CCCCATGCATTTTCCTTCTTGCCGGGATAGTGGCAGCTTCAAGGACAGCAGCTGA

MGNYKSRPTQTCTDEWKKKVNESYVITIERSEDDPRIKEKEFTEMRHIFGSDEAFSKVNLNYRTESGLSL
LHLCCICGGNQSHIRALMLKGLCPSLLTRNGFTALHLAVYKDSAELITSFLHSGGDVQQVGFGGLTALHI
AVIAGHLEAPDVLLQHGANVNVQDAVFFTPLHITVYYGHEQVTHLLLKFGADVNVSGEVGDRPLHLASAK
GFFNIAKLLMEDGNKADVNAQDNEDHDPLHFCSHFGHHEIMKYLLQSDSEVQPHVVNIYEDTPLHLACYN
GKFEVAKEIIQITGTESLTKENIFSETAFHSACTYGKSIDLVKFLLDQNVVNISHQGRDGHTGLHSACYH
GHIHLVQFLLDNGADMNLVACDPSRSSGEKNEQTCLMWAYEKGQDAIVTLLKHYKRPQDELPCNEYSRPG
GDGSYVSVPSPLGKIKNITKEKVDVLLLRARLPSHFHLQLSEIEFHEIIGSGSFGKVYKGRCRNKIVAIK
HC*

Figure S2 continued

D

Identities: 431/490(88%)
Positives: 453/490(92%)
Gaps: 1/490(0%)

```
Hgla   1  MGNYKSRPTQTCTDEWKKKVNESYVITIERSEDDPRIKEKEFTEMRHIFGSDEAFSKVNL  60
          MGNYKSRPTQTC+DEWKKKV+ESY I IER EDD +IKE EF E+RHIFGSDEAFS+V+L
Mmu    1  MGNYKSRPTQTCSDEWKKKVSESYAIIIERLEDDLQIKENEFQELRHIFGSDEAFSEVSL  60

Hgla  61  NYRTESGLSLLHLCCICGGNQSHIRALMLKGLCPSLLTRNGFTALHLAVYKDSAELITSF  120
          NYRTE GLSLLHLCC CGGN+SHIRALMLKGL PS LTRNGF ALHLAVYKDS ELITS
Mmu   61  NYRTERGLSLLHLCCACGGNKSHIRALMLKGLRPSRLTRNGFPALHLAVYKDSLELITSL  120

Hgla 121  LHSGGDVQQVGFGGLTALHIAVIAGHLEAPDVLLQHGANVNVQDAVFFTPLHITVYYGHE  180
          LHSG DVQQ G+GGLTALHIA IAGH EA +VLLQHGANVNVQDAVFFTPLHI  YYGHE
Mmu  121  LHSGADVQQAGYGGLTALHIAAIAGHPEAVEVLLQHGANVNVQDAVFFTPLHIAAYYGHE  180

Hgla 181  QVTHLLLKFGADVNVSGEVGDRPLHLASAKGFFNIAKLLMEDGNKADVNAQDNEDHDPLH  240
          QVT +LLKFGADVNVSGEVGDRPLHLASAKGFFNI KLL+E GNKADVNAQDNEDH PLH
Mmu  181  QVTSVLLKFGADVNVSGEVGDRPLHLASAKGFFNIVKLLVE-GNKADVNAQDNEDHVPLH  239

Hgla 241  FCSHFGHHEIMKYLLQSDSEVQPHVVNIYEDTPLHLACYNGKFEVAKEIIQITGTESLTK  300
          FCS FGHH I+ YLLQSD EVQPHV+NIY DTPLHLACYNG FEVAKEI+ +TGTESLTK
Mmu  240  FCSRFGHHNIVSYLLQSDLEVQPHVINIYGDTPLHLACYNGNFEVAKEIVHVTGTESLTK  299

Hgla 301  ENIFSETAFHSACTYGKSIDLVKFLLDQNVVNISHQGRDGHTGLHSACYHGHIHLVQFLL  360
          ENIFSETAFHSACTYGK+IDLVKFLLDQN VNI+H+GRDGHTGLHSACYHGHI LVQFLL
Mmu  300  ENIFSETAFHSACTYGKNIDLVKFLLDQNAVNINHRGRDGHTGLHSACYHGHIRLVQFLL  359

Hgla 361  DNGADMNLVACDPSRSSGEKNEQTCLMWAYEKGQDAIVTLLKHYKRPQDELPCNEYSRPG  420
          DNGADMNLVACDPSRSSGEK+EQTCLMWAYEKG DAIVTLLKHYKRPQDELPCNEYS+PG
Mmu  360  DNGADMNLVACDPSRSSGEKDEQTCLMWAYEKGHDAIVTLLKHYKRPQDELPCNEYSQPG  419

Hgla 421  GDGSYVSVPSPLGKIKNITKEKVDVLLLRARLPSHFHLQLSEIEFHEIIGSGSFGKVYKG  480
          GDGSYVSVPSPLGKIK++TKEK DVLLLRA LPS FHLQLSEIEFHEIIGSGSFGKVYKG
Mmu  420  GDGSYVSVPSPLGKIKSMTKEKADVLLLRAELPSRFHLQLSEIEFHEIIGSGSFGKVYKG  479

Hgla 481  RCRNKIVAIK  490
          RCRNKIVAIK
Mmu  480  RCRNKIVAIK  489
```

Figure S2 continued

E

ATGGGAAATTATAAATCTAGACCAACACAAACTTGTACTGATGAGTGGAAGAAGAAAGTCAATGAATCTT
ATGTTATTACAATAGAAAGATCAGAAGATGACCCGAGGATCAAGGAAAAAGAATTTACAGAGATGAGACA
TATTTTTGGGCCCCTGATGTGTTACTCCAACACGGAGCTAATGTCAGTGTTCAAGATGCTGTTTTTTTCA
CTCCATTGCACATTACAGTGTACTATGGGCATGAGCAGGTAACCCATCTCCTTTTGAAATTTGGTGCTGA
TGTAAATGTAAGTGGTGAAGTTGGGGATAGGCCTCTCCATCTGGCATCTGCAAAGGGATTCTTCAACATT
GCAAAACTCCTGATGGAAGATGGGAATAAAGCAGATGTGAATGCTCAGGATAATGAAGACCATGACCCAC
TGCATTTCTGTTCTCATTTTGGCCATCACGAGATAATGAAGTATCTGCTCCAAAGTGACTCGGAAGTTCA
GCCTCATGTTGTTAATATCTATGAAGACACTCCTTTGCACTTGGCATGCTACAATGGCAAATTTGAAGTT
GCTAAGGAGATCATTCAAATAACTGGAACAGAAAGTTTGACTAAGGAAAACATCTTTAGTGAGACAGCTT
TTCACAGTGCTTGTACCTATGGCAAGAGCATTGATCTGGTTAAATTTCTTCTTGATCAGAATGTGGTAAA
CATCAGTCACCAAGGAAGAGATGGGCACACAGGATTACACTCTGCTTGCTACCATGGTCACATTCACCTG
GTTCAGTTCTTACTGGACAATGGGGCTGATATGAATCTAGTTGCTTGTGATCCCAGCAGGTCTAGTGGTG
AAAAAAATGAGCAGACATGTTTGATGTGGGCTTATGAAAAAGGACAGGATGCCATTGTCACACTTCTGAA
GCATTATAAGAGACCCCAGGATGAACTGCCCTGTAATGAATATTCTCGTCCTGGAGGAGGTGCCCACTTT
TGCATTCACTTTTCATTGTTGTGATGCATGGCACCTAAAGAGAAGGTGGATGTTCTCCTCCTGAGGGCCA
GATTGCCTTCACATTTCCATCTTCAGCTCTCGGAAATTGAGTTCCATGAGATTATCGGCTCAGGTAACCT
AAAACACCACAGGTCTGCTTTAGTTTCATGAACCCGAGATAATCTGTGTTTATTTATTTTAAACAATTTC
AGGTTCTTTTGGGAAAGTATACAAAGGACGATGCAGAAATAAAATAGTGGCTATAAAACACTGTTGA

MGNYKSRPTQTCTDEWKKKVNESYVITIERSEDDPRIKEKEFTEMRHIFGPLMCYSNTELMSVFKMLFFS
LHCTLQCTMGMSR*

Figure S2 continued

F

```
Exon 5
          D  S  L  E  L  I  T  S  L  L  H  S  G  A  D  V  Q
Mmu ...agGATAGCCTGGAACTTATCACTTCACTGTTGCATAGCGGAGCAGATGTTCAG
Fdam...agGA-AGTGCAGAATCGATCACTTCCCTGCTGCACAGTGGGACTGATGAGCAG
Hgla...agGACAGTGCAGAACTGATCACTTCCTTCCTCCACAGTGGGGGTGATGTGCAG
          D  S  A  E  L  I  T  S  F  L  H  S  G  G  D  V  Q

             Q  A  G  Y  G  G  L  T  A  L  H  I  A  A  I  A
Mmu         CAAGCGGGATATGGCGGCCTCACAGCCCTCCACATCGCTGCAATAGCT...
Fdam        CAGGTGGGGTAAGGTGGCTTCTCTGCGCTCCACACTGCTGTCATAGCC...
Hgla        CAGGTGGGGTTCGGTGGCCTCACTGCACTCCACATTGCTGTCATCGCT...
             Q  V  G  F  G  G  L  T  A  L  H  I  A  V  I  A
```

F. damarensis exon 5

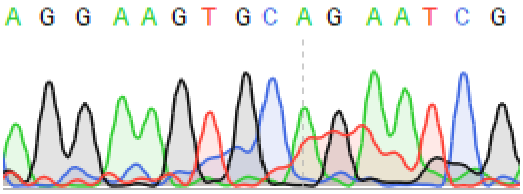

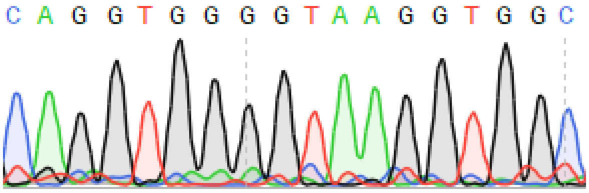


```
Exon 15
             S  F  G   K  V  Y  K  G  R  C  R  N  K  I  V  A  I
Mmu ...agGTTCTTTTGGG AAAGTCTATAAAGGGCGATGCAGAAATAAAATAGTGGCAATC...
Fdam...agGTTCTTTAGGGGAAGGTAAATAAAGGATGATGCAGGAATAAGATAGCGGCTATA...
Hgla...agGTTCTTTTGGG AAAGTATACAAAGGACGATGCAGAAATAAAATAGTGGCTATA...
             S  F  G   K  V  Y  K  G  R  C  R  N  K  I  V  A  I
```

F. damarensis exon 15

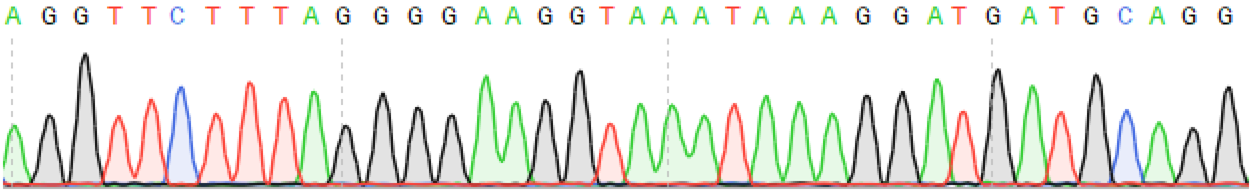

Supplement: S2 Fig — A. Alignment of portions of mouse exons 16, 20, and 21 with genomic DNA from the two mole-rat species (see S2 Table and S3 Table for coordinates of the mole-rat exon sequences), emphasizing the premature stop codons in exons 16 and 21 and the gap in exon 20 in H. glaber (in the center line), whereas F. damaraensis conserves the open reading frame in these regions. B. Sequence traces from H. glaber genomic DNA of the portions of exons 20 and 21 shown in panel A. C. Putative mRNA and translated reading frame as deduced from H. glaber genomic DNA, beginning with the ATG codon in exon 1 and ending with the TGA codon in exon 25. The premature stop codon in exon 16 is underlined and in red type. The putative translated product begins with the ATG codon in exon 1 and ends at the premature stop codon in exon 16. D. Alignment of the putative translated H. glaber (Hgla) Tnni3k protein with that of mouse (Mmu). E. Sequence of the Tnni3k cDNA as obtained experimentally from H. glaber ventricular mRNA, beginning with the ATG codon in exon 1 and ending with the premature stop codon in exon 16. The first in-frame stop codon is underlined and in red type. The translated product is shown below. F. Alignment of portions of mouse exons 5 and 15 with genomic DNA from the two mole-rat species, here with F. damarensis in the center line and organized to show the gaps and premature stop codons in both exons, whereas H. glaber conserves the open reading frame in these regions. Sequence traces of F. damarensis for these exon regions are shown below. (PDF) [file pgen.1008354.s002.pdf]
