# Supplementary material for: Tnni3k alleles influence ventricular mononuclear diploid cardiomyocyte frequency
Source: PLoS Genet. 2019 Oct 7;15(10):e1008354. doi: 10.1371/journal.pgen.1008354 (PMC6797218; doi:10.1371/journal.pgen.1008354)
Supplement: S1 Table — Accession numbers for Tnni3k or close homologs in human, mouse, sea urchin (S. purpuratus), golden apple snail (P. canaliculate) and Cailifornia two spot octopus (O. bimaculoides) are provided. (DOCX) [file pgen.1008354.s007.docx]

**S1 Table**: *Tnni3k* homologs in other species

| Common name | Species name | Superphylum | Phylum | Accession number |
| --- | --- | --- | --- | --- |
| Mouse | Mus musculus | Deuterostome | Chordata | NP_796040.3 |
| Human | Homo sapiens | Deuterostome | Chordata | NP_057062.1 |
| Sea Urchin | Strongylocentrotus purpuratus | Deuterostome | Echinodermata | XP_782227.3 |
| Golden apple snail | Pomacea canaliculate | Protostome | Mollusca | XP_025112879.1 |
| Cailifornia two spot octopus | Octopus bimaculoides | Protostome | Mollusca | XP_014790580.1 |
