# Supplementary material for: Tnni3k alleles influence ventricular mononuclear diploid cardiomyocyte frequency
Source: PLoS Genet. 2019 Oct 7;15(10):e1008354. doi: 10.1371/journal.pgen.1008354 (PMC6797218; doi:10.1371/journal.pgen.1008354)
Supplement: S2 Table — (DOCX) [file pgen.1008354.s008.docx]

**S2 Table**: Candidate assembly of *Tnni3k* gene in *Heterocephalus glaber* from NW_004624742.1 by homology to mouse gene

| exon | start | end |
| --- | --- | --- |
| 1 | 22423673 | 22423634 |
| 2 | 22423066 | 22422958 |
| 3 | 22406203 | 22406118 |
| 4 | 22404993 | 22404896 |
| 5 | 22388920 | 22388810 |
| 6 | 22318747 | 22318649 |
| 7 | 22314342 | 22314204 |
| 8 | 22308192 | 22308048 |
| 9 | 22307971 | 22307867 |
| 10 | 22304182 | 22304088 |
| 11 | 22303415 | 22303266 |
| 12 | 22290571 | 22290485 |
| 13 | 22289911 | 22289855 |
| 14 | 22288920 | 22288828 |
| 15 | 22288748 | 22288691 |
| 16 | 22288565 | 22288371 |
| 17 | 22287641 | 22287537 |
| 18 | 22229925 | 22229873 |
| 19 | 22229591 | 22229539 |
| 20 | 22225467 | 22225337 |
| 21 | 22214292 | 22214183 |
| 22 | 22184741 | 22184682 |
| 23 | 22182219 | 22182051 |
| 24 | 22134851 | 22134772 |
| 25 | 22131453 | 22131377 |
