# Supplementary material for: Tnni3k alleles influence ventricular mononuclear diploid cardiomyocyte frequency
Source: PLoS Genet. 2019 Oct 7;15(10):e1008354. doi: 10.1371/journal.pgen.1008354 (PMC6797218; doi:10.1371/journal.pgen.1008354)
Supplement: S3 Table — (DOCX) [file pgen.1008354.s009.docx]

**Supplemental Table S3**. Candidate assembly of *Tnni3k* gene in *F. damarensis* from NW_011046421.1 by homology to mouse gene

| exon | start | end |
| --- | --- | --- |
| 1 | 9488205 | 9488243 |
| 2 | 9488857 | 9488965 |
| 3 | 9500368 | 9500453 |
| 4 | 9501576 | 9501673 |
| 5 | 9513462 | 9513572 |
| 6 | 9563236 | 9563335 |
| 7 | 9567644 | 9567782 |
| 8 | 9573639 | 9573783 |
| 9 | 9573860 | 9573964 |
| 10 | 9577610 | 9577704 |
| 11 | 9578273 | 9578421 |
| 12 | 9591351 | 9591435 |
| 13 | 9591995 | 9592051 |
| 14 | 9592994 | 9593085 |
| 15 | 9593165 | 9593223 |
| 16 | 9593349 | 9593523 |
| 17 | 9594251 | 9594357 |
| 18 | 9657416 | 9657468 |
| 19 | 9657747 | 9657799 |
| 20 | 9662085 | 9662211 |
| 21 | 9673888 | 9673997 |
| 22 | 9699644 | 9699703 |
| 23 | 9702190 | 9702347 |
| 24 | 9742571 | 9742650 |
| 25 | 9745973 | 9746049 |
