# Supplementary material for: Tnni3k alleles influence ventricular mononuclear diploid cardiomyocyte frequency
Source: PLoS Genet. 2019 Oct 7;15(10):e1008354. doi: 10.1371/journal.pgen.1008354 (PMC6797218; doi:10.1371/journal.pgen.1008354)
Supplement: S4 Table — (DOCX) [file pgen.1008354.s010.docx]

**Supplemental Table S4**: Relatively common human *TNNI3K* kinase-domain variants (from ExAC)

| Variant | Consequence | Position in NP_057062.1 (human) | Position in NP_796040.3 (mouse) | Human allele frequency |
| --- | --- | --- | --- | --- |
| 1:74929170 T / C (rs3737564) | Ile > Thr | 686 | 685 | 0.01687 |
| 1:74905205 C / T (rs2274260) | Thr > Met | 637 | 636 | 0.002188 |
| 1:74836076 G / C (rs145260115) | Ser > Thr | 591 | 590 | 0.001142 |
| 1:74905291 G / A (rs148931950) | Ala > Thr | 666 | 665 | 0.0007842 |
| 1:74835130 G / T (rs34335537) | Val > Leu | 510 | 509 | 0.0004061 |
