# Supplementary material for: Tnni3k alleles influence ventricular mononuclear diploid cardiomyocyte frequency
Source: PLoS Genet. 2019 Oct 7;15(10):e1008354. doi: 10.1371/journal.pgen.1008354 (PMC6797218; doi:10.1371/journal.pgen.1008354)
Supplement: S5 Table — These data correspond to Figs. 2D, 3B, 5B and 5C. (DOCX) [file pgen.1008354.s011.docx]

**Supplemental Table S5:** Compilation of diploid nuclei percentages of mononucleated (Mono) and binucleated (Bi) cardiomyocytes in this study.

|  | Mouse Tnni3k genotype and CM type | | | | | | | | H glaber  CM type | |
| --- | --- | --- | --- | --- | --- | --- | --- | --- | --- | --- |
| Animal  number | +/- | | K489R/- | | -/- | | -/-, mCAT+ | |  |  |
|  | Mono | Bi | Mono | Bi | Mono | Bi | Mono | Bi | Mono | Bi |
| 1 | 72.7% | 96.8% | 79.2% | 92.3% | 61.9% | 88.1% | 69.2% | 87.1% | 21.4% | 75.2% |
| 2 | 77.8% | 83.9% | 57.1% | 90.1% | 66.7% | 84.9% | 84.2% | 87.7% | 28.6% | 63.4% |
| 3 | 65.2% | 84.9% | 59.3% | 88.7% | 76.2% | 74.1% | 62.5% | 81.5% | 32.1% | 78.9% |
| 4 | 52.4% | 78.2% | 72.0% | 89.7% | 53.6% | 81.4% |  |  | 8.8% | 67.6% |
| 5 | 60.9% | 85.4% | 66.7% | 82.8% | 70.0% | 78.6% |  |  |  |  |
| Average | 65.8% | 85.8% | 66.8% | 88.7% | 65.7% | 81.4% | 72.0% | 85.4% | 22.7% | 71.3% |
| Std Dev | 9.9% | 6.8% | 9.1% | 3.6% | 8.5% | 5.4% | 11.1% | 3.4% | 10.3% | 7.0% |
